# Remodeling nanodroplets into hierarchical mesoporous silica nanoreactors with multiple chambers

**DOI:** 10.1038/s41467-022-33856-y

**Published:** 2022-10-17

**Authors:** Yuzhu Ma, Hongjin Zhang, Runfeng Lin, Yan Ai, Kun Lan, Linlin Duan, Wenyao Chen, Xuezhi Duan, Bing Ma, Changyao Wang, Xiaomin Li, Dongyuan Zhao

**Affiliations:** 1 Department of Chemistry, Shanghai Key Laboratory of Molecular Catalysis and Innovative Materials, Laboratory of Advanced Materials, State Key Laboratory of Molecular Engineering of Polymers, iChEM (Collaborative Innovation Center of Chemistry for Energy Materials), Fudan University, Shanghai, 200433 P. R. China; 2 Academy of Medical Engineering and Translational Medicine, Medical College, Tianjin University, Tianjin, 300072 P. R. China; 3 State Key Laboratory of Chemical Engineering, East China University of Science and Technology, Shanghai, 200237 P. R. China

**Keywords:** Nanoparticles, Nanoparticles, Nanoparticle synthesis

## Abstract

Multi-chambered architectures have attracted much attention due to the ability to establish multifunctional partitions in different chambers, but manipulating the chamber numbers and coupling multi-functionality within the multi-chambered mesoporous nanoparticle remains a challenge. Herein, we propose a nanodroplet remodeling strategy for the synthesis of hierarchical multi-chambered mesoporous silica nanoparticles with tunable architectures. Typically, the dual-chambered nanoparticles with a high surface area of ~469 m^2^ g^−1^ present two interconnected cavities like a calabash. Furthermore, based on this nanodroplet remodeling strategy, multiple species (magnetic, catalytic, optic, etc.) can be separately anchored in different chamber without obvious mutual-crosstalk. We design a dual-chambered mesoporous nanoreactors with spatial isolation of Au and Pd active-sites for the cascade synthesis of 2-phenylindole from 1-nitro-2-(phenylethynyl)benzene. Due to the efficient mass transfer of reactants and intermediates in the dual-chambered structure, the selectivity of the target product reaches to ~76.5%, far exceeding that of single-chambered nanoreactors (~41.3%).

## Abstract (web summary)

Multi-chambered structures have attracted great attention due to their ability to create multifunctional partitions in different chambers. Here, the authors prepared mesoporous silica nanoreactors with hierarchical chambers for catalytic cascades.

## Introduction

Natural marvelous designs usually enlighten us to develop multifunctional architectures^1–5^ toward advanced technologies^6–12^. Typically, the compartmentalized multi-chambers within cells allow us to establish multifunctional partitions in different chambers (e.g., mitochondria, chloroplasts, etc.) and make multiple biochemistries perform without mutual interference^11,13^. Nanoarchitectures with multiple interior chambers can mimic the compartmentalized intracellular environment, allowing different chambers to perform distinct clearly defined functions and enabling certain substances to exchange through the boundaries^11,13–15^. It is crucial for expanding the application prospects of nanoparticles in the realms of nanoreactors, catalysis, drug delivery and so on^16–23^. A few other advantageous attributes associated with textural properties of multi-chambers include enlarged space for carrying incompatible or different components^11,22,24^, enhanced reactants accessibilities, and spatial confinement effects at specific locations^13^.

The implementation of all these advantages largely depends on the construction of multi-chambered nanostructures with tunable parameters, including number, distribution, composition, porosity, etc. To date, various strategies have been excavated to pursue these goals^7,25,26^. For example, the representative hard-templating routes^27^, which involve solid surface modification, target material coating, and template removal, have been widely used for synthesis porous core-multishelled (layer-to-layer cavity) nanomaterials with various compositions. Due to its simplicity, it can also be extended to the preparation of other multi-chambered nanoparticles with yolk-multishell^28^ or groove-cavity structures^29^. Unfortunately, the multiple spaces in these core@shell-based multi-chambered structures are either nested or interrelated, which cannot provide truly independent spaces for different functional entities. Recently, Chen et al.^30,31^ proposed an approach for the interconnection of independent hollow units from individual to multi-compartmented carbon-based vessels via a liquid template strategy. This method demonstrated the flexibility of droplets in shape control, especially in the regulation and manipulation of droplets during the process^32,33^. Nevertheless, current methods still lack effective design over the spatial isolation of multiple functional entities, and the shells formed in the multi-compartment structure are comprised of dense, solid parts, further restricting the storage and efficient diffusion of guest molecules^34–38^. The fabrication of mesoporous-shelled nanostructures with a tunable number and distribution of chambers as well as multifunctional (magnetic, catalytic, optic, etc.) synergetic configurations still remains a challenge, which is crucial for the construction of multifunctional mesoporous nanoreactors for cascade reactions^29,39–44^.

Herein, a nanodroplet remodeling strategy is demonstrated to synthesize hierarchical multi-chambered mesoporous silica nanoparticles with tunable chamber numbers (from single to tri-chambered nanoarchitectures). Typically, the dual-chambered mesoporous silica nanoparticles show a calabash shape that is clearly different from the tadpole-like morphology reported previously in our group^45^. The calabash-like nanoparticles with a body length of 482–565 nm exhibit two hierarchical parts with an outer diameter of 192 ± 32 nm for the first level (bottom) and 133 ± 28 nm for the second level, respectively. The mesoporous silica nanoparticles possess an interconnected hollow chamber (inner diameter: 141 ± 33 nm for the first level and 102 ± 26 nm for the second level, respectively), pore size of 2.64 nm, and a high surface area of ~469 m^2^ g^−1^. Meanwhile, the inner cavity can be manipulated from enclosed to open and the center distance between the two adjacent chambers can also be tuned from 235 ± 38 to 475 ± 47 nm. Besides, this nanodroplet remodeling strategy can also facilitate the selective assembly of functional units (Fe_3_O_4_, Pd, Pt, etc.) in the multi-chambered nanoparticles. As a proof of concept, the dual-chambered nanoreactors were constructed with the Au nanocrystals mainly riveted at the first chamber and Pd nanocrystals anchored in the second open cavity for the cascade synthesis of 2-phenylindole from 1-nitro-2-(phenylethynyl)benzene. Benefiting from the different spatial distribution of the catalytic active centers to a certain extent, the dual-chambered nanoreactor exhibits good catalytic performance in the cascade synthesis of 2-phenylindole with a high selectivity of 76.5%, which is 1.85 times higher than that of the single-chambered nanoreactor. Meanwhile, the simulation results demonstrate that the dual-chambered structure can guarantee efficient mass transfer of reactants and intermediates, thereby facilitating the improvement of the selectivity to 2-phenylindole product.

## Results

### Calabash-like mesoporous silica nanoparticles

The multi-chambered mesoporous silica nanoparticles were fabricated via a nanodroplet remodeling strategy in a water-in-oil nanoemulsion system. The spherical water nanodroplets in *n*-pentanol were stabilized by hexadecyltrimethylammonium bromide (CTAB) surfactant. Then, the silicate oligomers produced by the hydrolysis of tetraethyl orthosilicate (TEOS) were adsorbed on the oil–water interface by electrostatic interaction. The growth of silica shells triggered the interface migration and further transformed the nanodroplets into an elongated ellipsoid shape^45^. Afterward, a new solvent (e.g., tetrahydrofuran (THF)) with a certain distribution coefficient (*K*_ow_: 0.94) in the oil–water system was added to remodel the nanodroplets. Because one side of the droplets is covered by the formed rigid silica, the diffusion of THF into the water phase caused the volume expansion of the nanodroplets from the exposed side, resulting in the formation of the dual-chambered mesoporous nanoparticles.

**Fig. 1:**
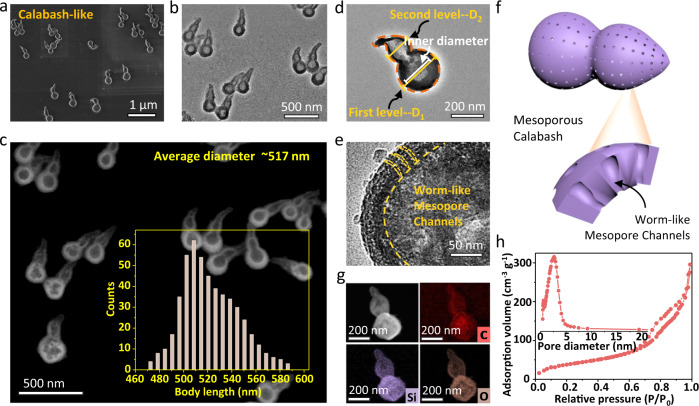
Calabash-like mesoporous silica nanoparticles. **a** SEM, **b**, **d**, **e** TEM, and **c** dark-field TEM images of the dual-chambered mesoporous silica nanoparticles with different magnifications; Inset of **c** is the corresponding distribution histograms of the particle body lengths. **f** The structural model, and **g** element mappings of the mesoporous nanoparticle; **h** Nitrogen sorption isotherms, and the pore size distribution (inset of **h**) of the mesoporous silica nanoparticle. A total of 504 nanoparticles are analyzed. Source data are provided as a Source Data file.

The obtained mesoporous silica nanoparticles present a calabash-shaped morphology with two ellipsoidal nodes (Fig. 1a). The body length of the mesoporous nanoparticle ranges from 482 to 565 nm. A dual-chambered structure can be clearly observed in this mesoporous nanoparticle (Fig. 1b, c), and the outer diameter of the nanoparticles is measured to be 192 ± 32 nm for the first level and 133 ± 28 nm for the second level, respectively. The transmission electron microscopy (TEM) image clearly demonstrates the enclosed hollow structure of the nanoparticle, and the inner diameter of the cavity also decreases from 141 ± 33 nm for the first level to about 102 ± 26 nm for the second level (Fig. 1d). The wormlike mesopores can clearly be observed in the shell of the silica nanoparticle (Fig. 1e, f and Supplementary Fig. 1). Different elements (including C, Si, and O) can be identified by the energy dispersive spectrometer mapping, which further demonstrates the uniform deposition of silicate oligomers at the interface (Fig. 1g). Nitrogen adsorption-desorption isotherms of the obtained hollow mesoporous nanoparticles display characteristic type-IV curves with distinguishable capillary condensation at *P*/*P*_0_ = 0.6–0.9, demonstrating the open mesopore channels. The Brunauer–Emmett–Teller (BET) surface area and pore volume are calculated to be ~469 m^2^ g^−1^ and 0.63 cm^3^ g^−1^, respectively (Fig. 1h). The pore size obtained by the Barrett–Joyner–Halenda (BJH) method is centered at 2.64 nm.

### Hierarchical multi-chambered mesoporous nanoparticles

**Fig. 2:**
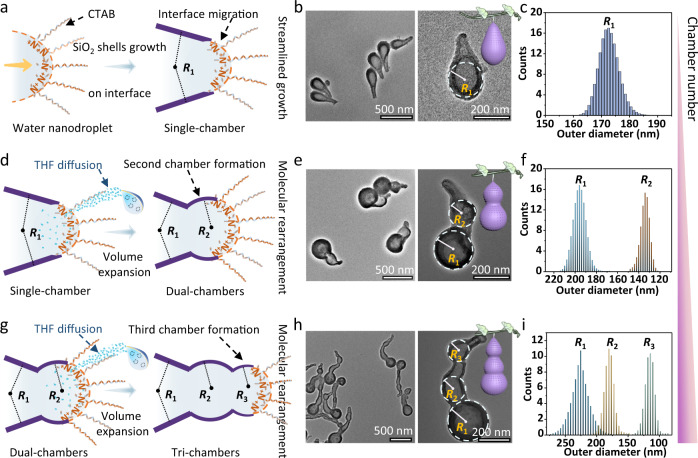
Hierarchical multi-chambered mesoporous silica nanoparticles with tunable chamber numbers. **a**, **d**, **g** The illustration of the nanodroplet remodeling process. By controlling the number of the THF additions, the chamber numbers can be tuned from one to three. **b**, **e**, **h** TEM images and **c**, **f**, **i** the distribution histograms of the outer diameter of the hierarchical multi-chambered mesoporous silica nanoparticles with a variable number of chambers: **b**, **c** single-chamber, **e**, **f** dual-chambers, and **h**, **i** tri-chambers. Insets in **b**, **e**, and **h** are the structural models of mesoporous silica nanoparticles. Source data are provided as a Source Data file.

Without the addition of THF solvent, the streamlined mesoporous nanoparticles with a single chamber can be formed (Fig. 2a–c). The number of the ellipsoidal nodes can be further tuned from two to three by regulating the number of THF additions (Fig. 2d, e, g, h and Supplementary Fig. 2). It is noteworthy that the curvature radius (*R*_*n*_, *n* stands for different levels) of the subsequently increased ellipsoidal chamber decreases gradually with the increase of the chamber numbers. For instance, the calculated curvature radius of the tri-chambered nanoparticles gradually decreases from 21.5 ± 5.2 to 8.9 ± 3.6 nm, which is consistent with the change in outer diameter (Fig. 2f, i and Supplementary Fig. 3). Furthermore, the addition of THF has no obvious effect on the deposition of surfactant/silicate composite micelles at the interface, so the mesoporous nanoparticles with a different number of chambers all show wormlike mesoporous channels (Supplementary Fig. 4). However, the resulting fourth chamber became indistinct after the third addition of THF, most likely due to the slower diffusion speed induced by the reduced THF concentration difference between the inside and outside of the droplet, resulting in smaller subsequent bulges (Supplementary Fig. 5). Besides, the volume of the added THF has great effects on the size of the newly formed chamber. Obviously, the average size of the nanodroplets gradually increases from 194 to 296 nm with the increase of the additional amount of THF, indicating the volume expansion of the nanodroplets (Supplementary Fig. 6a, b). As the amount of THF increases from 0.05 to 0.20 mL, the outer diameter of the second chamber gradually increases from 20 ± 8 to 135 ± 30 nm (Supplementary Fig. 7).

Meanwhile, it is found that the diffusion process of THF from oil to water phase does not influence the positive surface charge of the nanodroplets because of electroneutral THF molecules. However, as THF diffuses from the oil phase to the aqueous droplet, the zeta potential value of the nanodroplet first decreases and then gradually increases to a stable value, indicating that the diffusion reaches a new equilibrium state (Supplementary Fig. 8). Nevertheless, when CTAB concentration is relatively low (<4.64 mmol/mL), the insufficient stabilization of the droplets leads to the formation of indistinct cavity structure (Supplementary Fig. 9a, b). With the increase of CTAB concentration (8.27 mmol/mL), the chamber structure of the obtained nanoparticles gradually becomes clear (Supplementary Fig. 9c). The size of the first chamber increases to 137 ± 31 nm, and the second cavity also becomes obvious with the addition of THF. However, no further significant change in the cavity size is observed, and the aggregation occurs between the formed multi-chambered silica particles when CTAB concentration is too high (12.18 mmol/mL, Supplementary Fig. 9d).

In addition, the influence of the diffusion behavior of different solvents (e.g., THF, DMSO, etc.) on the chamber structure is further studied (Supplementary Fig. 10). Obviously, although the same volume of solvent is added into the emulsion (without the addition of TEOS), the increase in the droplet size after THF addition (0.10 mL) is greater than that of butanol, but slightly smaller than that of DMSO, which can be attributed to the different oil–water partition coefficient of different solvents (Supplementary Fig. 6c). However, when a more hydrophilic solvent is used, such as water, since it is insoluble in the oil phase, the subsequently added water can disperse into smaller nanodroplets after entering the system. Part of the small nanodroplets can adhere to the exposed point of the original droplets (some small droplets exist independently) and continue to complete an anisotropic growth, resulting in a solid rod connected with a hollow cavity. By contrast, for more lipophilic solvents, such as hexanol (distribution coefficient *K*_ow_: ~1.82, Supplementary Table 1), since less part enters the water phase, the mutation caused by volume expansion becomes less obvious, resulting in a smaller second node.

### Tunable openings and chamber distribution

**Fig. 3:**
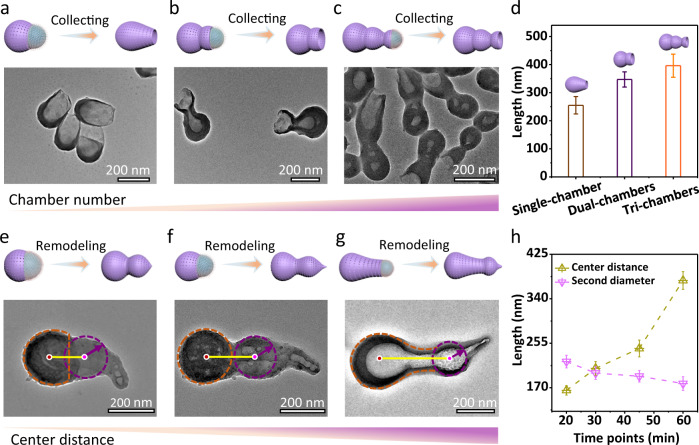
Hierarchical multi-chambered mesoporous nanoparticles with tunable opening and chamber distribution. **a**–**c** Structural models, TEM images, and **d** the corresponding distribution histograms of the body length of the multi-chambered mesoporous nanoparticles with tunable openings; **a** single-chamber, **b** dual-chambers, **c** tri-chambers. **e**–**g** Structural models and TEM images of the dual-chambered nanoparticles with tunable chamber distance. **h** The relative changes of center distance and the diameter of the second chamber. Error bars represent standard deviation. Source data are provided as a Source Data file.

In this nanoemulsion system, stopping the reaction (varying the reaction time) before the silica shells completely cover the droplets, a single-chambered structure with an opening of 60–105 nm can be formed (Fig. 3a and Supplementary Fig. 11a). Unlike single-chambered nanoparticles, the dual-chambered structure exhibits a streamlined head connected with another opened cavity (Fig. 3b and Supplementary Fig. 11b). After the first addition of THF, the length of the second chamber (distance between the junction of the two chambers and the opening) can be adjusted from 50 ± 22 to 380 ± 35 nm as the reaction time increases from 40 to 60 min (Supplementary Fig. 12). The tri-chambered nanoparticles can be obtained after the droplet being reshaped twice, which is composed of a two-chambered calabash and a streamlined tail with an opening of 105 ± 37 nm (Fig. 3c and Supplementary Fig. 11c). The average body length also increases from 255 ± 31 nm to 396 ± 44 nm with the chamber number expands from one to three (Fig. 3d).

Moreover, taking the dual-chambered structure as an example, adding THF prematurely (when the first section only grows ~20 min) results in a reduced center distance (L) between the two adjacent chambers (Fig. 3e). In contrast, the delay of THF addition can induce the increase of L to 377 ± 17 nm (Fig. 3f–h and Supplementary Figs. 13 and 14). Similarly, in the tri-chambered architecture, the center distance between the second and third chamber can also be adjusted from 235 to 375 nm (Supplementary Fig. 15). Since the droplets are gradually elongated as the silica shell grows, the addition of THF at different time points can adjust the center distance between the adjacent chambers.

### Selectively in situ assembly of different functional units

**Fig. 4:**
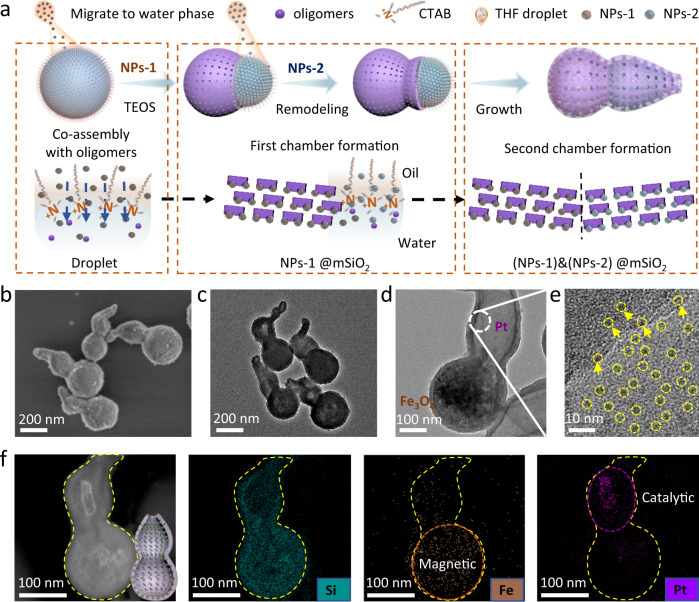
Selectively in situ assembly of different functional units. **a** Schematic illustration of the selective in situ loading of hydrophilic nanoparticles in different chambers. **b** SEM, **c**–**e** TEM, and **f** element mapping images of the dual-chambered mesoporous nanoparticle with Pt loaded in the upper and magnetic Fe_3_O_4_ nanoparticles anchored in the bottom chambers. Insets in **f** are the structure model of the nanocrystal-loaded mesoporous dual-chambered nanoparticles.

Taking the hydrophilic metal nanoparticles (Fe_3_O_4_, Pt, etc.) as an example, the direct addition of magnetic Fe_3_O_4_ nanoparticles (~20 nm) in the initial stage can make it encapsulate in the first cavity, and the second addition of Pt-containing THF solution can anchor Pt nanocrystals (~4 nm) in the second cavity while reshaping the nanodroplets (Fig. 4a–e). Elemental mappings further demonstrate the maximum spatial isolation of the Fe_3_O_4_ and Pt nanocrystals in the bottom and upper chambers, respectively, which is consistent with their sequence of addition (Fig. 4f). Moreover, this stepwise encapsulation strategy shows good scalability, other functional nanoparticles, e.g., catalytic Au (~6 nm) or Pd (~4 nm), up-conversion luminescent NaYF_4_ nanoparticles (~22 nm), etc., can also be implanted into specific locations (Supplementary Figs. 16 and 17). The successful isolation and coordination of the functional entities in the multiple chambers is due to the fact that hydrophilic nanoparticles in the THF can migrate into the water phase and adsorb at the water–oil interface, thus crosslinking with the silicate oligomers and further implanting on the inner walls, thereby avoiding excessive mutual crosstalk and leaching. In addition, functional nanoparticles (such as Au) can also be in situ fabricated in the chamber due to the presence of abundant mesopores (Supplementary Fig. 18).

### Dual-chambered nanoreactors

**Fig. 5:**
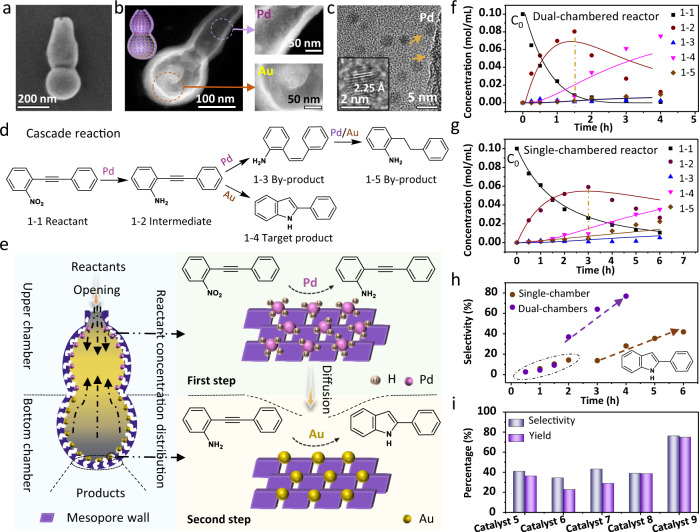
Dual-chambered mesoporous silica nanoreactors. **a** SEM, **b** HAADF-STEM, and **c** high-resolution TEM (HRTEM) images of the obtained dual-chambered mesoporous silica nanoreactors by selectively loading Au and Pd nanocrystals in the bottom and upper cavities of reactors, respectively. The inset in **b** is the 3D structural model and inset in **c** is the HRTEM image of the Pd nanocrystals. **d** Schematic illustration, **e** concentration distribution of reactants, and **f** kinetic plots of the cascade reaction in the dual-chambered nanoreactor (Au in the bottom chamber, Pd in the upper chamber). **g** Kinetic plots of the cascade reaction in the single-chambered nanoreactor (Au and Pd loaded in the same chamber). **h** The selectivity to 2-phenylindole as a function of time. **i** The yield and selectivity of 2-phenylindole under different catalysts: mixed-Au and Pd in single-chambered silica particles (**Catalyst 5**), mixed-Au and Pd nanocrystals (**Catalyst 6**), mixed-catalyst 1 and 2 (**Catalyst 7**), mixed-Au and Pd on dense silica spheres (**Catalyst 8**), and spatial separation of Au and Pd in dual-chambered silica particles (**Catalyst 9**), respectively. Note: in the cascade synthesis of 2-phenylindole, the autoclave was purged with hydrogen gas at least three times and the H_2_ pressure was set to 1.5 MPa. The temperature was kept at 80 °C. Source data are provided as a Source Data file.

As a proof of concept, we constructed an opened dual-chambered mesoporous nanoreactor with Au nanocrystals (~4 nm, ~2.45 wt%) mainly trapped at the bottom cavity and Pd nanocrystals (~2.5 nm, ~1.65 wt%) implanted in the upper chamber (Fig. 5a–c and Supplementary Fig. 19) for the cascade synthesis of 2-phenylindole from 1-nitro-2-(phenylethynyl)benzene, which are highly value-added intermediates in fine chemicals and pharmaceuticals^46–50^. As a control, a single-chambered nanoreactor was also designed with two kinds of nanocrystals loaded in the same chamber (the cavity volume and the catalyst content are basically the same as that of the dual chambers) (Supplementary Figs. 20 and 21). The synthesis of 2-phenylindole from 1-nitro-2-(phenylethynyl)benzene involves the reduction of nitro groups and the subsequent cyclization reaction. Conventionally, this catalytic process is expected to proceed sequentially to avoid the generation of various by-products, e.g., 2-(phenylethynyl)aniline and 2-phenethylaniline. In this regard, such a dual-chambered system enables maximum spatial isolation of two kinds of catalytic active sites in one single nanoparticle, thus facilitating the cascade synthesis of 2-phenylindole (Fig. 5d, e).

The catalytic activities of the monometallic catalysts, including Pd in the dual-chambered silica (denoted as **Catalyst 1**), Au in the dual-chambered silica (**Catalyst 2**), bare Pd nanocrystals (**Catalyst 3**), and bare Au nanocrystals (**Catalyst 4**), were firstly tested. It is found that the monometallic Pd in the dual-chambered silica (**Catalyst 1**) catalyst can give raw reactant 1-nitro-2-(phenylethynyl)benzene in ~100% conversion, and almost no target 2-phenylindole product can be detected (Supplementary Fig. 22), reflecting that the Pd has the high catalytic activity of hydrogenation reaction for the generation of intermediate product, but almost no catalytic activity of cyclization reaction for the generation of target product. On the contrary, the individual Au in the dual-chambered silica (**Catalyst 2**) provides only ~48.2% conversion of 1-nitro-2-(phenylethynyl)benzene raw reactant, whereas the conversion of 2-(phenylethynyl)aniline intermediate (used as reactant) to 2-phenylindole reaches as high as ~90%, demonstrating that Au is mainly active for the cyclization reaction. The catalytic properties of bare Pd (**Catalyst 3**) and bare Au (**Catalyst 4**) are similar to those of **Catalyst 1** and **2**, respectively, but the difference is that the corresponding conversions are reduced (Supplementary Fig. 23). Considering the catalytic efficiencies of different metal catalysts (Au/Pd) for hydrogenation and cyclization reactions, the co-existence of Au and Pd may play a synergistic catalytic effect.

Indeed, in the Pd and Au spatially isolated dual-chambered architecture, the 2-(phenylethynyl)aniline intermediate attains a maximum concentration of 0.082 mol L^−1^ only at 1.5 h (Fig. 5f and Supplementary Fig. 24), in comparison with that of 0.059 mol L^−1^ at 3 h for single-chambered nanoreactors (Fig. 5g), indicating the relatively higher catalytic efficiency of the first step in the dual-chambered structure, which may be attributed to the efficient diffusion and mass transfer of reactants. Meanwhile, a small amount of 2-styrylaniline by-products are produced with the increase of the intermediate concentration in the initial reaction stage of both nanoreactors, which is finally converted into 2-phenylethylaniline by-products. Moreover, the kinetic plots further show a rapid drop in the intermediate concentration accompanied by a sudden increase in 2-phenylindole product (from the second to the fourth hour in Fig. 5f), suggesting the efficient progress of the subsequent cyclization reaction in the dual-chambered nanoreactor.

However, although the yield of the target product 2-phenylindole increases to 36.6% for the Au and Pd mix-loaded single-chambered silica particles (**Catalyst 5**), the co-loading of Au and Pd in one chamber cannot effectively suppress the occurrence of side reactions, so the selectivity of the target product is only 41.3%. Nonetheless, the selectivity is further reduced to 34.7% for the mixed-Au and Pd nanocrystals without encapsulation (**Catalyst 6**), indicating the necessity of silica encapsulation. Similarly, both mixed-catalyst 1 and 2 (**Catalyst 7**, 29.2% yield of target product), and Au-Pd mixed-loaded dense silica (**Catalyst 8**, 38.9% yield of target product) catalysts can also increase the yield of the target product to a certain extent (Supplementary Fig. 25), but the by-products are also increased simultaneously, resulting in the selectivity remaining basically unchanged compared with that of Au and Pd co-stored single-chambered catalyst (Supplementary Table 2). These results are most likely due to the fact that the Pd and Au active centers are either too far apart (**Catalyst 6** and **7**) or excessively crosstalk (**Catalyst 5** and **8**), resulting in few intermediates that can diffuse to the independent Au active sites, thus increasing the production of by-products. By contrast, the selectivity of the target product 2-phenylindole significantly increases from 41.3% (single-chamber) to ~76.5% for the dual-chambered nanoreactor with a certain degree of spatial separation of Au and Pd active centers (Fig. 5h and Supplementary Fig. 26). Meanwhile, the maximum yield of the target product 2-phenylindole (~75.2%) in dual-chambered nanoreactor is also far beyond that of other types of catalysts (Fig. 5i), further highlighting the advantage of spatial isolation of dual-catalysts in the dual-chambered nanoreactor for cascade reactions.

Moreover, the catalytic stability measurements show that the activity of the catalyst in the dual-chambered nanoreactors exhibits a relatively slow decay rate in the first five cycles. The corresponding initial reaction rate decays gently from ~102.78 for the first cycle to ~99.57 mmol h^−1^ mmol_pd_^−1^ for the fifth cycle (Supplementary Fig. 27). After five rounds of catalytic reactions, Pd/Au is still well anchored in the cavity, and no obvious Pd/Au leaching is detected in the reaction solution, further illustrating the stability of the nanocrystals in the chamber structure. However, the decay rate is accelerated after five cycles. After the 10th cycle, the corresponding initial reaction rate is reduced to 57.81 mmol h^−1^ mmol_pd_^−1^ and the yield of 2-phenylindole product is only 31.3%. The decay of catalyst activity may be caused by the slight aggregation of Pd nanocrystals and very small amount (~0.11%) of Pd leaching due to slight etching of the SiO_2_ framework after 10 cycles, as well as catalyst poisoning caused by the adsorption of nitrogenous species (Supplementary Fig. 28).

**Fig. 6:**
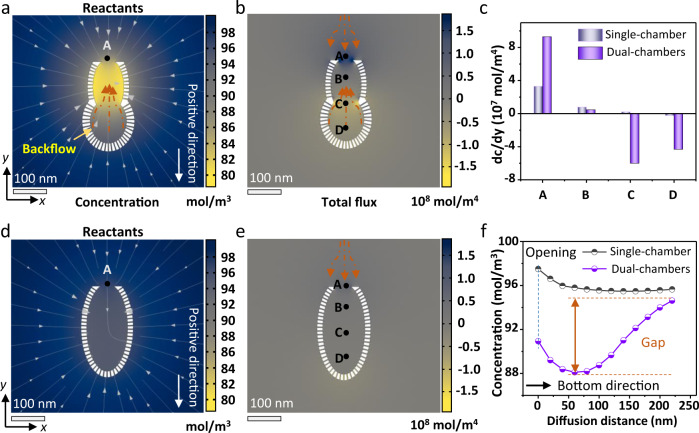
Finite element analyses of the mass transfer in the nanoreactors with different morphologies. **a**, **d** The simulated transient-state concentration gradient of the reactants in different nanoreactors at 20 µs. **b**, **e** The corresponding total flux profiles of reactants in single- and dual-chambered nanoreactors at 20 µs. Position A is the opening of the nanoreactor, Position B is the center location of the upper chamber, Position C is the middle of the whole cavity, and Position D is the center location of the bottom chamber. **c** The calculated transient-state concentration gradient of the reactants at 20 µs at different positions of the nanoreactors. **f** The concentration distribution as a function of diffusion distance (different position) at 20 µs. The diffusion of reactants to the bottom chamber is specified as the positive direction. The simulations were performed in a time-dependent mode at a microsecond scale due to the rapid evolution of physical fields. Source data are provided as a Source Data file.

To further evaluate the significant difference in mass transfer, finite element analysis method was applied to simulate the transient-state concentration gradient distribution of reactants and intermediates in the nanoreactors with different chamber structures. The simulations were performed in a time-dependent mode at a microsecond scale due to the rapid evolution of physical fields. It is observed that the rapid depletion of the 1-nitro-2-(phenylethynyl)benzene reactants in the upper chamber results in a rapid decrease in the concentration of the reactants, thereby generating a large concentration gradient at the A position (the opening of the nanoreactor) (Fig. 6a b). The calculated concentration gradient at A position of the dual-chambered nanoreactor is 2.29 × 10^7^ mol m^−4^, much higher than that of single-chambered nanoreactor (0.63 × 10^7^ mol m^−4^ at A position) (Fig. 6c), indicating that a large concentration difference is generated between the inner cavity and the outside. This ensures the rapid entry of the reactants into the upper chamber from the opening and further reflects the efficient production of 2-(phenylethynyl)aniline intermediates. In addition, the rapid depletion of reactants in the upper chamber can simultaneously produce a large concentration difference from the bottom chamber. Driven by the resultant concentration gradient (position C and D in Fig. 6c), the reactants in the bottom chamber can flow back into the upper chamber (Supplementary Movie 1). Therefore, compared with the single-chambered nanoreactor (Supplementary Movie 2), the dual-chambered nanoreactor can simultaneously realize the supply of reactants from the bottom chamber to the upper chamber (Fig. 6d–f). This process can also drive the efficient diffusion of the 2-(phenylethynyl)aniline intermediates generated in the upper cavity to the bottom chamber (Supplementary Movie 3), which facilitates the subsequent cyclization to produce 2-phenylindole. Besides, the external intermediates can also directly re-diffuse into the bottom chamber with Au active sites through the mesopore shells, and the internal intermediates can maintain a high diffusion flux to the bottom chamber, both of which are crucial to reduce the generation of by-products and improve the selectivity of the target product (Supplementary Figs. 29 and 30).

## Discussion

**Fig. 7:**
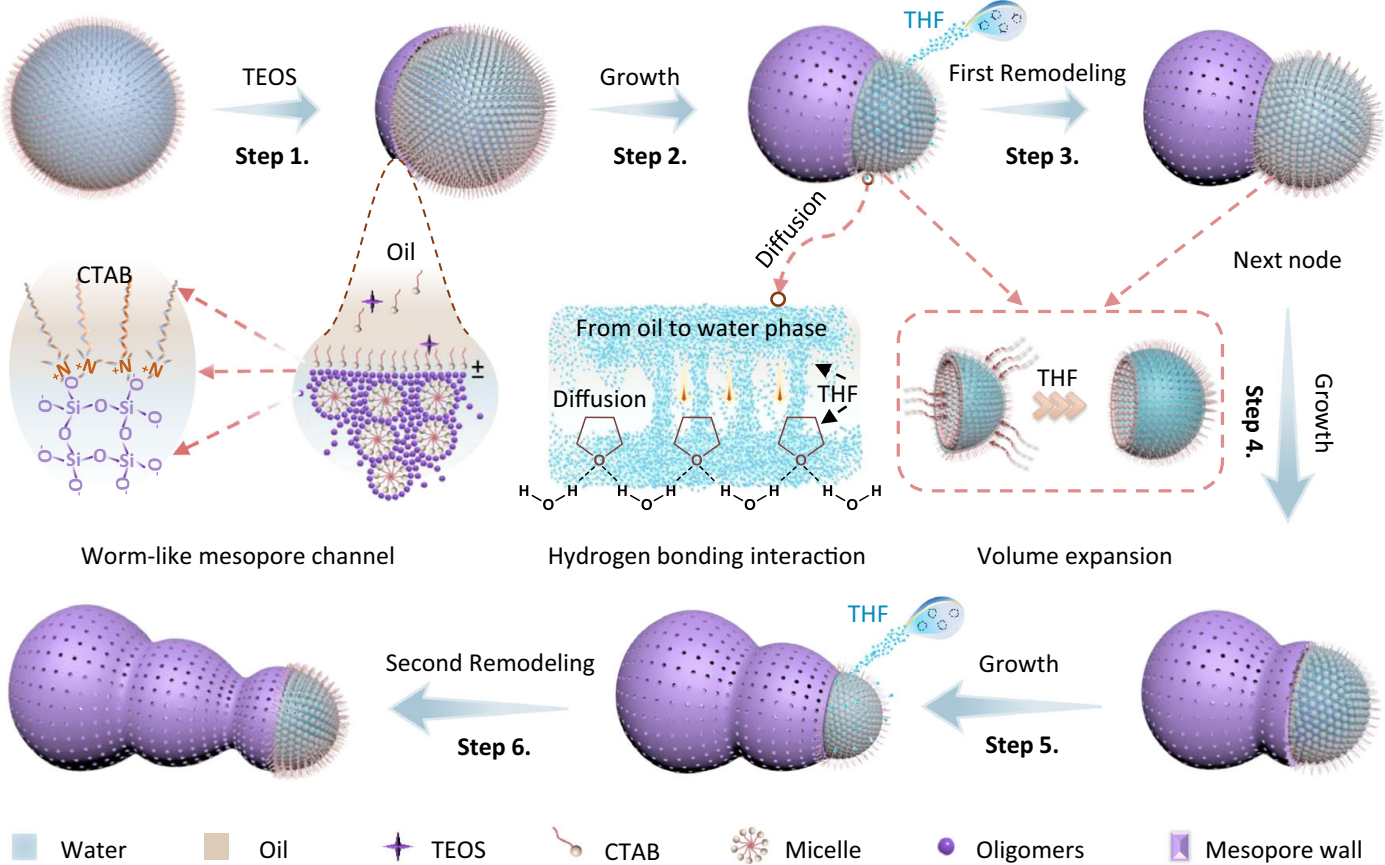
The illustration of the nanodroplet remodeling strategy. The formation process of the multi-chambered mesoporous silica nanoparticles. The multi-chambered nanoparticles can be fabricated by adding solvents with certain oil–water distribution coefficients (e.g., THF) at different stages.

On the basis of the above results, a nanodroplet remodeling strategy is proposed for the fabrication of the multi-chambered mesoporous nanoparticles with tunable cavity numbers. In the water-in-oil nanoemulsion system, the droplets are stabilized by the cationic surfactant CTAB, and the excess surfactants can migrate into the water phase form the micelles (Fig. 7, Step 1). The negatively charged silicate oligomers produced by the hydrolysis of TEOS are continuously deposited at the interface under the attraction of positively charged surfactant molecules^45,51^. In addition, the silica oligomers further crosslink, polymerize and co-assemble with the surfactant/silicate composite micelles in the water phase, triggering the dynamic migration of the oil–water interface, resulting in the anisotropic growth of the mesoporous silica shells, which also leads to the formation of the wormlike mesopore channels (Step 2, Supplementary Fig. 31a, b)^45^. When the silica shells grow for 0.5 h, a new solvent (THF, hexane, hexanol, etc.) with a certain distribution coefficient in the oil–water system is added. Due to the different distribution ratio of THF in oil and water, partial THF molecules diffuse into the water phase driven by the intermolecular hydrogen bond interactions (Supplementary Fig. 32), which further results in the volume expansion of the water nanodroplet (Step 3, Supplementary Fig. 31c, d). Because one side of the droplets is confined by the rigid silica shells, the volume expansion can only occur on the exposed side of the droplets to form an abrupt turn at the oil–water interface, thus generating a second chamber due to the volume expansion of the droplets. This process also drives the movement of the surfactant molecules at the oil–water interface, thus becoming new sites for the deposition of the silicate oligomers. As the continuous deposition of the silicate oligomers at the newly formed oil–water interface, a second node is generated (Step 4, 5, Supplementary Fig. 31e, f). Similarly, the second addition of THF solvent can further cause the outward expansion of nanodroplets, resulting in the formation of the third chamber (Step 6).

In addition, the multi-chambered structure cannot only enhance the catalytic performance through back-flowing and efficient diffusion of reactants, but also reduce the excessive mutual crosstalk and influence between different catalytic active sites through the spatial isolation of different catalysts in the different chambers. Specifically, the enhanced catalytic performance of the dual-chambered nanoreactors in this work with respect to the single-chambered one may be derived as follows: compared with the single-chambered nanoreactor, the dual-chambered design cannot only ensure the rapid arrival of reactants to the catalytic active centers in the upper chamber (Pd) but also induce the back-flow of the reactants from the bottom chamber to the upper chamber, which is beneficial to improve the reaction efficiency of the former reaction of the cascade catalytic reaction. On the other hand, during the catalytic reaction, the generated small amount of by-products (2-styrylaniline in this case) can further occupy Pd or Au active sites for the next hydrogenation reaction, thus producing another by-product (in this case, 2-phenethylaniline). In the Pd and Au co-loaded single-chambered nanoreactors, the by-products and the intermediates may simultaneously compete for the active sites of Au, which is not conducive to the cyclization reaction of the intermediates (the latter reaction of the cascade catalytic reaction), resulting in a significant increase in the by-products (especially, 2-phenethylaniline) and a decrease in the target products (2-phenylindole in our case). In contrast, in the dual-chambered nanoreactors with the spatial isolation of two active centers, the produced small amount of the by-products (e.g., 2-styrylaniline in this case) can be directly transformed into another by-product under the catalysis of Pd in the upper chamber, limiting its competition with intermediates for the Au active sites in the bottom chamber. In addition, the dual-chambered design can also enable the outside intermediates to directly reenter the bottom chamber with Au active sites through the mesoporous shells. The intermediates diffused to the Au active sites are subsequently consumed for the production of the target product (2-phenylindole), which effectively inhibits the side reaction and improves the selectivity of the target product.

In summary, a family of mesoporous nanoparticles with tunable number and distribution of chambers have been fabricated via the nanodroplet continuous remodeling strategy. This approach enables the manipulation of nanodroplets at the single-droplet level, which is achieved by regulating the number of solvents (with certain oil–water distribution coefficients) additions. The dual-chambered nanoparticles present a calabash-like morphology with a body length of 482–565 nm (outer diameter: 192 ± 32 nm for the first and 133 ± 28 nm for the second level, respectively) and high surface areas (~469 m^2^ g^−1^). The inner diameter of the two adjacent chambers decreases from 141 ± 33 to 102 ± 26 nm and the center distance between the two chambers can be tuned from 235 to 375 nm. Such a nanodroplet remodeling strategy can facilitate the selective assembly of functional units in the multi-chambered nanoparticles. Nanoparticles (Fe_3_O_4_, Pd, Pt, Au, etc.) with different functions can be carried in different spatial regions of the dual-chambered mesoporous nanoarchitectures, thus effectively preventing the excessive mutual interference between different functional units. The simulation results confirm that a certain degree of spatial isolation of dual-catalysts in the dual-chambered mesoporous nanoreactor and the presence of mesoporous shells can guarantee the efficient diffusion of reactants and intermediates, thus reaching a high selectivity of 76.5% in the cascade synthesis of 2-phenylindole. Such multi-chambered nanoparticles with tunable number and distribution of chambers as well as selective functionalization have great potential to become a promising multifunctional platform not only for nanoreactors but also for cargo delivery, diagnosis, and sensing.

## Methods

### Chemicals

CTAB, polyvinylpyrrolidone (PVP, molecular weight 55,000 g mol^−1^), sodium citrate monohydrate (99%), sodium citrate dihydrate (99%), and TEOS were purchased from Aladdin (Shanghai, China). 1-Octadecene (ODE), oleylamine (OAm), trioctylphosphine (TOP), palladium acetylacetonate (Pd(acac)_2_), platinum acetylacetonate (Pt(acac)_2_), dimethyl formamide (DMF), THF, ethyl acetate (EtOAc), *n*-pentanol, ammonium hydroxide (NH_3_·H_2_O 28 wt%), anhydrous ethanol, iron (III) chloride hexahydrate (99%), iron (II) chloride tetrahydrate (99.95%) were purchased from Sinopharm (Shanghai, China). All chemicals were used as received without further purification. Deionized water was used for all experiments.

### Synthesis of multi-chambered mesoporous silica nanoparticles

The multi-chambered mesoporous silica nanoparticles were synthesized by using the nanodroplet remodeling strategy^45,52^. In general, 1.0 mL of ethanol, 0.12 mL of aqueous sodium citrate solution (0.15 M), and 0.10 mL of NH_3_·H_2_O (28 wt%) were mixed with 10.0 mL of *n*-pentanol under continuous sonication to form a uniform emulsion. Then, 40.0 mg of CTAB and 1.0 g of PVP were added to the above-mentioned emulsion in sequence under continuous sonication until completely dissolved. After sonicating for 10 min, 0.15 mL of TEOS was added. Subsequently, 0.10 mL of THF was added when the reaction solution standing for 0.5 h at room temperature. The dual-chambered mesoporous silica nanoparticles could be formed after the reactions standing for another 1.5 h. The final products were centrifuged (10,000 r/min, centrifugal force, 5900 g) and washed three times with ethanol. Then, the collected products were extracted with 0.1 M of HCl in ethanol solution at 60 °C for 12 h twice to remove the residual surfactants.

For the regulation of the chamber numbers, the above reactions were performed similarly except that increasing the number of THF additions. In detail, after the first addition, the same amount of THF was added after 20 min to obtain tri-chambered mesoporous silica nanoparticles.

### Synthesis of hydrophilic Fe_3_O_4_

Hydrophilic Fe_3_O_4_ nanoparticles were prepared based on the method reported in the literature^53^. In brief, 3.40 g of FeCl_3_·6H_2_O and 0.80 g of FeCl_2_·4H_2_O were added to 40.0 mL of water in a three-necked flask and heated to 90 °C under a nitrogen atmosphere. After quickly adding 5.0 mL of NH_3_·H_2_O (28 wt%) and stirring for 0.5 h, 4.0 mL of sodium citrate aqueous solution (2.0 M) was added to the above solution, and the reaction was continued for another 2 h. The Fe_3_O_4_ nanoparticles were washed twice with deionized water and redispersed in 40.0 mL of deionized water.

### Synthesis of Pd nanocrystals

Pd nanocrystals were synthesized by using the procedures reported previously^54^. In a typical process, 40.0 mL of ODE, 2.20 mL OAm and 1.20 mL of TOP were mixed with 0.30 g of the Pd(acac)_2_ in a three-neck flask. After being evacuated for 0.5 h at room temperature, the mixture was further evacuated at 100 °C for another 0.5 h. Then, the reaction flask was flushed with argon and heated to 280 °C (15 °C/min) until the solution turned orange. After 30 min, the solution was cooled to room temperature. The nanoparticles were isolated by the addition of isopropanol (100 mL) and centrifugation (10,000 rmp, 6 min, centrifugal force, 5900 g), and finally redispersed in cyclohexane (2 mg/mL). For the removal of surface ligands, 3.0 mL of the above-mentioned Pd-containing cyclohexane solution, 3.0 mL of DMF were mixed with 120 mg of NOBF_4_. Shaking the mixture for 5 min, then let it stand for layering. After the upper cyclohexane was removed, ethanol was added for centrifugal washing (12,000 rmp, 10 min, centrifugal force, 8496 g). Finally, the collected products were redispersed into ethanol.

### Synthesis of hydrophilic Au nanoparticles

Au nanoparticles were prepared in a manner similar to the procedures reported previously^55,56^. In detail, 0.10 mmol of HAuCl_4_·3H_2_O was added to 100 mL of deionized water, and then the mixture was heated to approximately 95 °C under stirring. Then, 2.82 mL of sodium citrate aqueous solution (0.20 M) was rapidly added to the above solution with stirring. The solution was heated for 35 min until the color became deep red-burgundy, and then cooled to room temperature.

### Anchoring Au and Pd nanocrystals in different chambers

For the loading of Au nanocrystals into the bottom of the dual-chambered mesoporous silica nanoparticles, 0.10 mL of the Au nanocrystal-containing THF solution (~4.0 mg/mL) was added to 10.0 mL of *n*-pentanol at the initial stage. All the other reaction conditions were consistent with that of the synthesis of the dual-chambered mesoporous silica nanoparticles. Then, after reacting for 0.5 h, 0.10 mL of the Pd-containing THF solution (2.0 mg/mL) was added to the above solution. By reacting for another 0.5 h, the dual-chambered mesoporous silica nanoparticles with Au anchored in the bottom and Pd nanocrystals loaded in the upper cavity could be obtained. The Fe_3_O_4_/Pt-loaded nanoparticles could be obtained by replacing the Au nanocrystal-containing THF solution with an equal amount of Fe_3_O_4_/Pt-containing THF solution.

### In situ preparation of Au in dual-chambered silica

1.0 mL of 1.0 mg mL^−1^ obtained silica nanoparticles was centrifuged and redispersed in a solution containing 2.5 mL of 2.5 mM HAuCl_4_ and 2.5 mL of 5.0 mM sodium citrate. After the above solution was sonicated for 20 min, the superfluous solution was removed by centrifugation at 6000 r for 5 min. The reaction was continued for 24 h until the mesoporous nanoparticles gradually changed from white to dark purple, demonstrating the formation of Au nanoparticles. To speed up the reaction process, 100 μL of 20 mM NaBH_4_ solution was added to the sample after centrifugation (10,000 r/min, centrifugal force, 5900 g), and the reaction could be completed within 0.5 h. The final sample was washed with water and collected by centrifugation.

### Catalysis reaction

#### The preparation of 1-nitro-2-(phenylethynyl)benzene reagent

Phenylacetylene (123 mg, 1.20 mmol), 1-iodo-2-nitrobenzene (249 mg, 1.00 mmol), Pd(PPh_3_)_2_Cl_2_ (35 mg, 0.050 mmol), and CuI (6.0 mg, 0.030 mmol) were mixed with 5.0 mL of THF at room temperature. Then, the solution of ethanolamine (305 mg, 5.0 mmol) in water (4.0 mL) was added to the above mixture. The obtained mixture was heated to 80 °C for 12 h, then cooled to room temperature and diluted with water (10.0 mL) and EtOAc (10.0 mL). The aqueous phase was extracted with an additional EtOAc (20.0 mL). The combined organic phases were washed with brine (25.0 mL). The resultant organic phase was dried over Na_2_SO_4_, filtered, and concentrated in vacuo. The residue was purified by flash column chromatography (SiO_2_, EtOAc:Hex = 1:50) to give the desired products as a red-brown oil.

#### Cascade synthesis of 2-phenylindole

The cascade synthesis of 2-phenylindole was carried out in 25.0 mL of autoclave equipped with a Teflon tube (Parr). 5.0 mL of absolute ethyl alcohol, 0.50 mmol of 1-nitro-2-alkenylbenzenes, and 20.0 mg of the mesoporous silica nanoparticles loaded with Pd and Au catalysts obtained above were added into the autoclave. The autoclave was purged with hydrogen gas at least three times and the final reaction pressure was set to 1.5 MPa. The stirring speed was fixed at 600 rpm (centrifugal force, 4.248 g), and the temperature was kept at 80 °C. After the mixture was stirred for 6 h, the H_2_ was carefully released. The filtrate was concentrated in vacuo to give the desired product as an off-white solid.

### Simulation of concentration distribution in the nanoreactor

COMSOL Multiphysics software was used to simulate the transient-state concentration gradient distribution of intermediate products. The model was established on the transport of diluted species interface. The reaction order and rate in the nanoreactors with different chamber structures were estimated by fitting the kinetic curves. Therefore, the inflow boundary condition at the reaction surface was used. The contour of the nanoreactor was built using the elliptic equation for the head and Bézier curve for the body. The data of concentration gradient were obtained after a running time of 10^−5^ s and collected at the middle position of the chamber.

### Data collection and analysis

To measure the structural parameters of the dual-chambered nanoparticles, we examined ten randomly selected locations on the TEM/SEM grid to analyze the products. To be more accurate, the measurements were also taken from distinct batches of samples. We counted more than 500 particles and estimated the body length. Other parameters were estimated in the same way, and the corresponding number of statistical particles was also shown in the figure caption. In addition, the description of size values was shown in a relative range, or the type of mean ± standard deviation, etc.

### Characterizations

Nitrogen adsorption-desorption isotherms were performed at 77 K with a Micromeritcs Tristar 3020 analyzer (USA). Before measurement, the samples were degassed in a vacuum at 180 °C for at least 8 h. The BET specific surface areas were calculated from the adsorption data in the relative pressure (*P/P*_*0*_) ranging from 0.02 to 0.2. The pore size distribution curves were derived from the adsorption branches of the isotherms using the BJH model. Pore volumes were calculated from the adsorption branches of isotherms, and the total pore volume was estimated from the adsorbed amount at the relative pressure (*P/P*_*0*_) of 0.995. The field-emission scanning electron microscope (FESEM) observations were taken on a Hitachi FE-SEM-4800 microscope operating at 20 kV without any metal coating. TEM measurements were taken on a JEOL JEM-2100F microscope (Japan) operated at 200 kV. The samples used for TEM and FESEM analyses were dispersed in ethanol and dried on amorphous carbon-coated Cu grids.

### Reporting summary

Further information on research design is available in the Nature Research Reporting Summary linked to this article.

## Supplementary information

Supplementary Information

Description of Additional Supplementary Files

Supplementary Movie 1

Supplementary Movie 2

Supplementary Movie 3

Reporting Summary

## Data Availability

Data supporting the findings of this study are available within the article and the associated Supplementary information section. The source data underlying Figs. 1–6 and Supplementary figure are provided in a source data file. Source Data are provided with this paper.

## Source data

Source Data
