# Supplementary material for: Remodeling nanodroplets into hierarchical mesoporous silica nanoreactors with multiple chambers
Source: Nat Commun. 2022 Oct 17;13:6136. doi: 10.1038/s41467-022-33856-y (PMC9576742; doi:10.1038/s41467-022-33856-y)
Supplement: Supplementary file 1 — Supplementary Information [file 41467_2022_33856_MOESM1_ESM.pdf]

Supplementary Information

Remodeling nanodroplets into hierarchical mesoporous silica nanoreactors with multiple chambers

Yuzhu Ma[1#], Hongjin Zhang[2#], Runfeng Lin[1], Yan Ai[1], Kun Lan[1], Linlin Duan[1], Wenyao Chen[3], Xuezhi Duan[3], Bing Ma[1], Changyao Wang[1], Xiaomin Li[1*], Dongyuan Zhao[1*]

[1]Department of Chemistry, Shanghai Key Laboratory of Molecular Catalysis and Innovative Materials, Laboratory of Advanced Materials, State Key Laboratory of Molecular Engineering of Polymers, *i*ChEM (Collaborative Innovation Center of Chemistry for Energy Materials), Fudan University, Shanghai, 200433, P. R. China.
[2]Academy of Medical Engineering and Translational Medicine, Medical College, Tianjin University, Tianjin 300072, China.
[3]State Key Laboratory of Chemical Engineering, East China University of Science and Technology, Shanghai 200237, China.
[#]These authors contributed equally: Yuzhu Ma, Hongjin Zhang.
[*]Corresponding authors' E-mails: dyzhao@fudan.edu.cn; lixm@fudan.edu.cn

Supplementary Figures

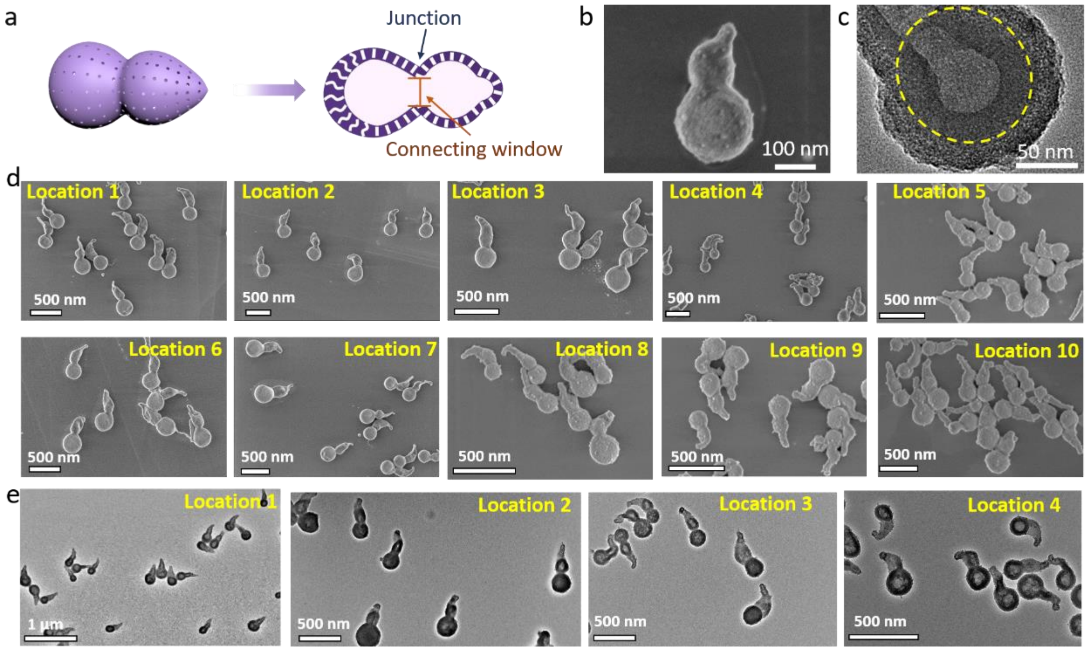

Supplementary Figure 1. Dual-chambered calabashes. (a) Structural models, (b, d) SEM, and (c, e) TEM images of the dual-chambered mesoporous silica nanoparticles prepared by the nanodroplet remodeling approach with the addition of THF for once (the images were taken from different locations of one sample). The calabash-like structure can be clearly observed and the silica shells are composed of disordered worm-like mesopore channels. In addition, more than ten locations were randomly selected on the SEM/TEM grid to analyze the obtained products. We counted more than 500 particles and estimated the structural parameters. For example, the body length of the mesoporous nanoparticle was measured to be 482-565 nm.

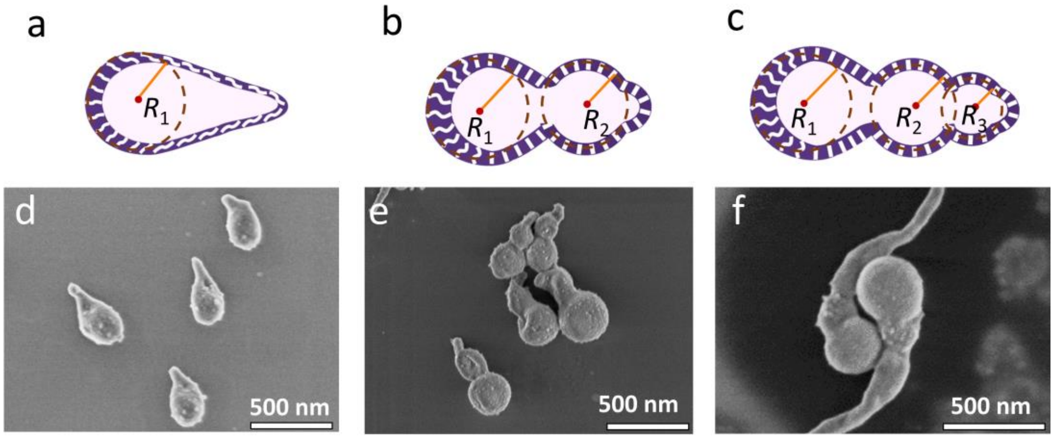

Supplementary Figure 2. Adjustable chamber numbers. (a-c) Structural models and (d-f) SEM images of the mesoporous silica nanoparticles prepared by the nanodroplet remodeling approach with different number of remodelings (*i.e.*, different number of THF additions); (a, d) without remodeling (single-chamber), (b, e) remodeling once (dual-chambers), and (c, f) remodeling twice (tri-chambers). Without the addition of THF solvent, the single-chambered mesoporous silica nanoparticles could be formed. The dual-chambered nanoparticles could be obtained by controlling the nanodroplet to be reshaped once (adding THF once). Similarly, the tri-chambered nanoparticles could be formed after the second addition of THF solvent.

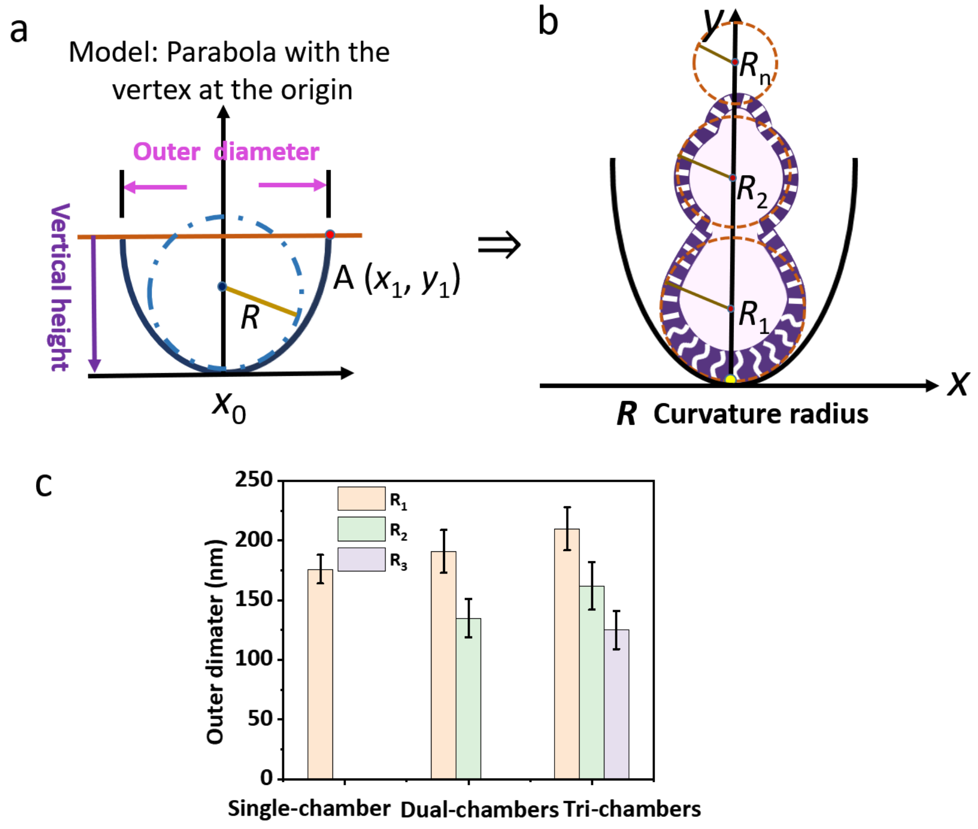

Supplementary Figure 3. Curvature radius calculation method. (a) Calculation of curvature radius; (b) Two-dimensional (2-D) curve simulation based on the shape of the particle shape. A parabolic model was used to calculate the curvature radius of the mesoporous silica nanoparticles.

The curvature radius was calculated as following:

$$r = \frac{1}{K} = \frac{(1+y'^2)^{\frac{3}{2}}}{|y''|}$$

Where K is the surface curvature (nm^{-1}); y' is the first derivative of the curve; y'' is the second derivative of the curve; r is the curvature radius (nm). The black curve represents the simulated parabola, the brown line represents the curvature radius, the red circle represents the curvature circle; (c) The distribution histograms of the outer diameter of the multi-chambered mesoporous silica nanoparticles. Obviously, the outer diameter of the tri-chambered structure gradually decreases with the increase of the chamber numbers. 267 nanoparticles are analyzed. Source data are provided as a Source Data file.

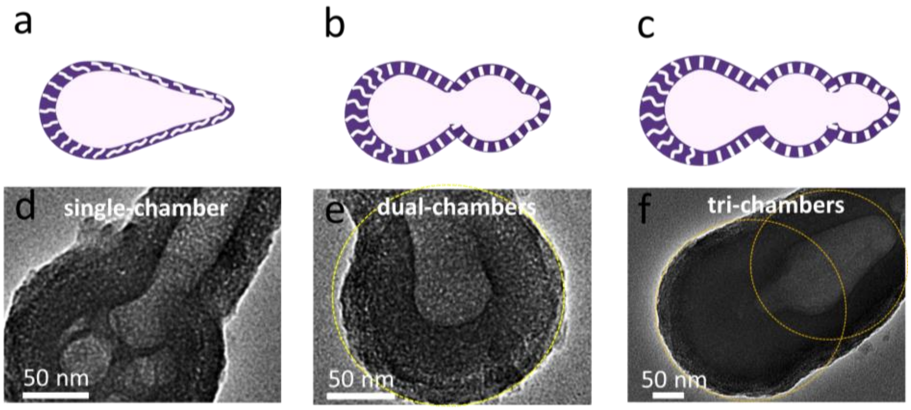

Supplementary Figure 4. Worm-like mesopore channels in nanoparticles with different chamber numbers. (a-c) Structural models and (d-f) TEM images of the mesoporous silica nanoparticles prepared by the nanodroplet remodeling approach with different number of remodelings (*i.e.*, different THF addition times), (a, d) without remodeling (single-chamber), (b, e) remodeling once (dual-chambers), and (c, f) remodeling twice (tri-chambers), showing that the silica shells are all composed of the worm-like diverging mesopore channels.

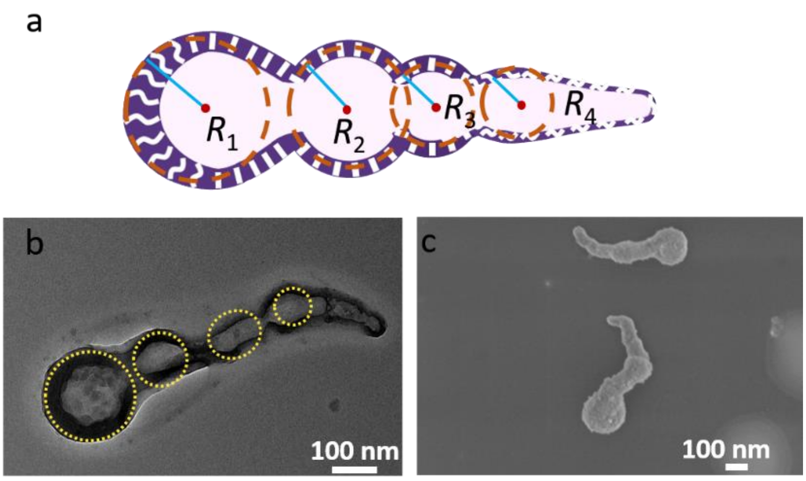

Supplementary Figure 5. Tetra-chambered mesoporous nanoparticles. (a) The structural model, (b) TEM, and (c) SEM images of the tetra-chambered mesoporous silica nanoparticles prepared by the multiple nanodroplet remodeling approach after adding THF for three times. The resulting fourth chamber became indistinct after the third addition of THF, most likely due to the slower diffusion speed induced by the reduced THF concentration difference between the inside and outside of the droplet, resulting in smaller subsequent bulges. Subsequent exploration of the penta-chamber design also yielded similar results.

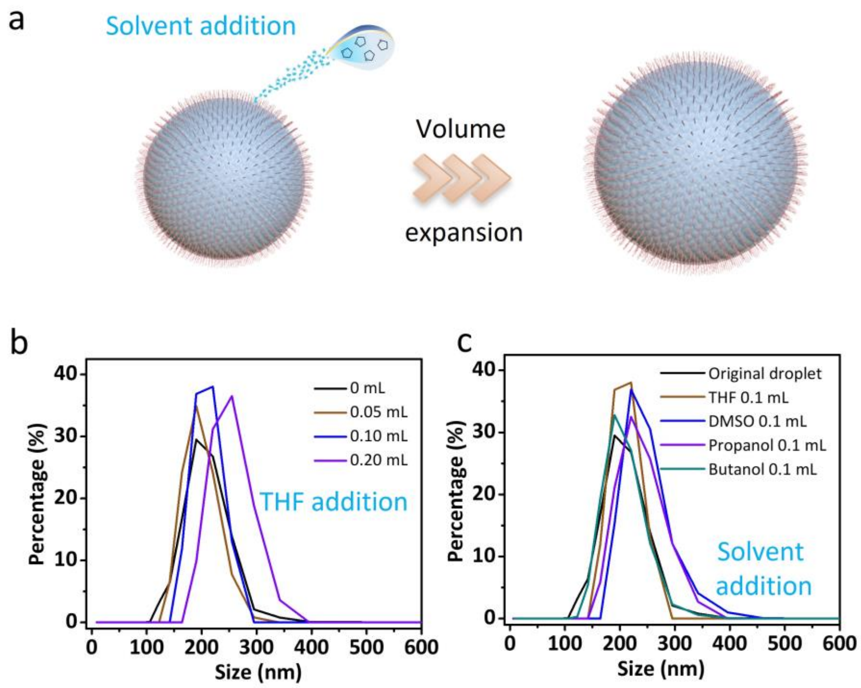

Supplementary Figure 6. Influence of added solvents on the size of nanodroplets. (a) The illustration of the volume expansion process of the nanodroplets after the addition of THF. (b) The variation of the nanodroplet size as the increase of addition amount of THF from 0 to 0.20 mL. (c) Changes in the nanodroplet size after the addition of 0.1 mL of solvents with different oil-water partition coefficients. Obviously, when the same volume of solvent is added to the emulsion (without adding TEOS), the size of the nanodroplets increases differently. Taking THF as an example, dynamic light scattering (DLS) tests show that the average size of the nanodroplets gradually increases from 194 to 296 nm with the addition amount of THF increase from 0 to 0.2 mL, indicating the effective diffusion of THF into the water droplet. Meanwhile, the increase in the droplet size after THF addition is greater than that of butanol, but smaller than that of DMSO, which can be attributed to the different oil-water distribution coefficients of different solvents. Similarly, DMSO can also form hydrogen bonds with water molecules. After the addition DMSO, the size of the nanodroplets also increases, and finally a dual-chambered structure can also be formed. Source data are provided as a Source Data file.

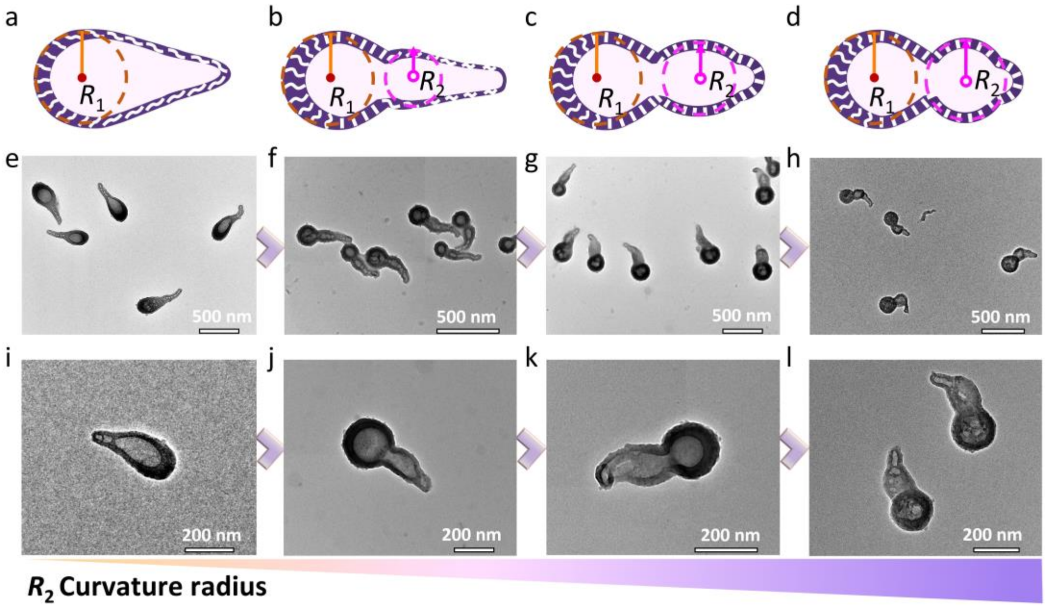

Supplementary Figure 7. Influence of THF amount on the chamber structure. (a-d) Structural models and (e-l) TEM images of the silica nanoparticles prepared by the nanodroplet remodeling approach with different volumes of THF solvent, (a, e, i) 0 mL, (b, f, j) 0.05 mL, (c, g, k) 0.10 mL, and (d, h, l) 0.20 mL. Obviously, without the addition of THF solvent, the single-chambered mesoporous silica nanoparticles can be formed. As THF volume increases from 0.05 to 0.20 mL, the cavity structure gradually changes from single chamber to a connected dual-chambered nano-architecture. The outer diameter of the second chamber gradually increases from 25±8 to 135±30 nm, indicating that the THF volume is crucial for the formation of the second chamber.

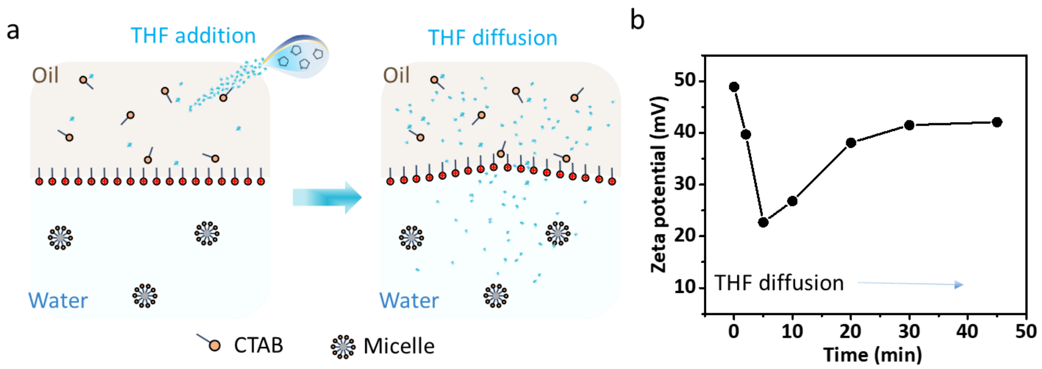

Supplementary Figure 8. Influence of THF diffusion process on the zeta potential of nanodroplets. (a) The schematic illustration of THF diffusion from oil to water phase, (b) The zeta potential changes of the nanodroplet with time during the THF diffusion process. As THF diffuses from the oil phase to the water droplet, the zeta potential value of the nanodroplet first decreases and then gradually increases to a stable value, indicating that the diffusion reaches a new equilibrium state. This is probably because the initial diffusion of THF into the water phase leads to the volume expansion of the droplets, which disrupts the charge balance at the interface, thus making the decrease of the surface potential. With the rearrangement and replenishment of CTAB molecules, the interface charge gradually increases to a new equilibrium value. Source data are provided as a Source Data file.

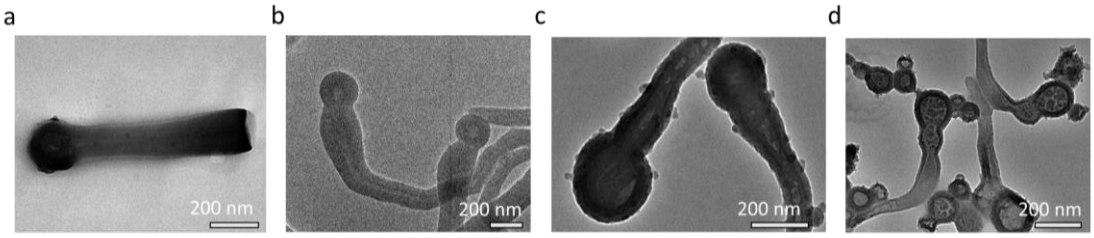

Supplementary Figure 9. **Influence of CTAB concentration on chamber structure.** TEM images of the samples obtained with different concentrations of surfactant CTAB: (a) 0 mmol/mL; (b) 4.64 mmol/mL; (c) 8.27 mmol/mL; and (d) 12.18 mmol/mL. Without CTAB, nanoparticles with essentially no internal cavity were formed. When the CTAB concentration is relatively low (<4.64 mmol/mL), the size of the first chamber increased to ~25 nm, but the second chamber structure formed after THF addition was inconspicuous. With the increase of CTAB concentration (8.27 mmol/mL), the cavity structure of the obtained nanoparticles gradually became clear. The size of the first chamber increased to 137±31 nm, and the second cavity also became obvious with the addition of THF. However, when the CTAB concentration was too high (12.18 mmol/mL), the cavity size did not further change significantly, but aggregation was generated between the formed multi-chambered silica particles.

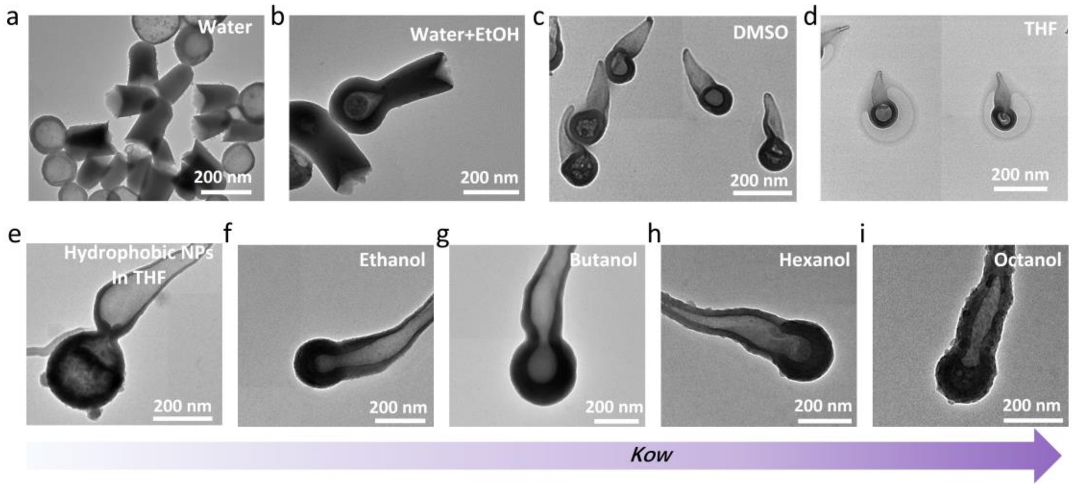

Supplementary Figure 10. Influence of different solvents on second chamber. (a-i) TEM images of the silica nanoparticles prepared by the nanodroplet remodeling approach based on the solvents with different oil-water partition coefficients. In the water-in-oil emulsion system, the influence of the diffusion behavior of different solvents (THF, hexane, *etc*.) on the chamber structure was further studied. For example, since water is insoluble in the oil phase, the subsequently added water can disperse into smaller droplets after entering the system. Part of the small nanodroplets can be attached to the exposed side of the original droplets (some small droplets exist independently) and continue to complete an anisotropic growth. In contrast, after the addition of a solvent with a certain oil-water partition coefficient (THF, for example), it can diffuse into the water phase driven by intermolecular hydrogen bonds and cause the expansion of the water droplets. However, due to the different oil-water distribution coefficients of different solvents, the structures of the second chamber formed are correspondingly different. For example, the use of a relatively more hydrophilic solvent (except water) makes the formed second chamber more pronounced, while using a more lipophilic solvent results in a relatively smaller outer diameter of the second chamber. Particularly, after dispersing some small hydrophobic nanoparticles in the THF solvent, the corresponding node caused by using the same volume of the solvent is more obvious, probably due to the migration of small nanoparticles to the oil-water interface leading to more THF entering the droplet. It has been demonstrated that the nanoparticles can migrate to the water/oil interface and further stabilize the droplets to form the Pickering emulsion.[7-9]

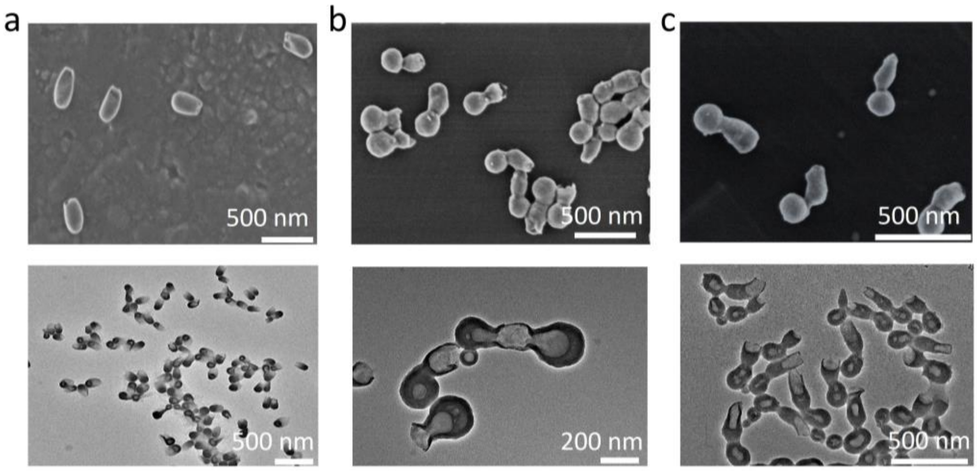

Supplementary Figure 11. Variable chamber numbers with controlled opening. (a-c) SEM and TEM images of the mesoporous silica nanoparticles prepared by the nanodroplet remodeling approach at different reaction times, (a) without remodeling-45 min, (b) remodeling once-50 min, and (c) remodeling twice-60 min. Without nanodroplet remodeling, a single-chambered structure with an opening of 60-105 nm could be formed in 45 min. After the first remodeling (reaction for another 20 min), the obtained dual-chambered structure exhibited a streamlined head connected with another opened tail (diameter: 90±22 nm). The tri-chambered nanoparticles obtained after the nanodroplets being reshaped twice (total reaction time: 60 min) were composed of a dual-chambered calabash and a streamlined tail with an opening of 85±33 nm.

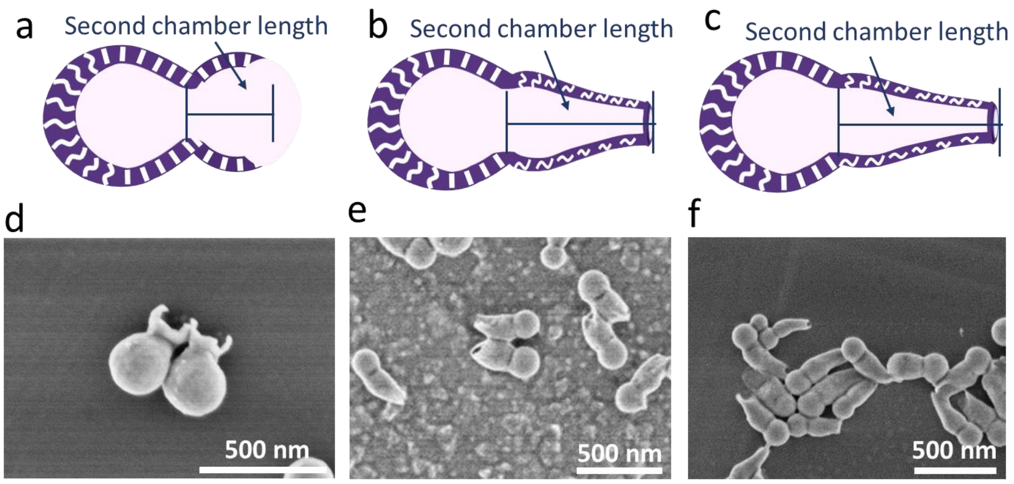

Supplementary Figure 12. Adjustable length of second chamber. (a-c) Structural models, (d-f) SEM images of the dual-chambered mesoporous silica nanoparticles obtained with different reaction times at (a, d) 40 min, (b, e) 50 min, and (c, f) 60 min. After the first addition of THF, the lengths of the second opened cavity could be adjusted from 50±22 to 380±35 nm with the reaction time increased from 40 to 60 min.

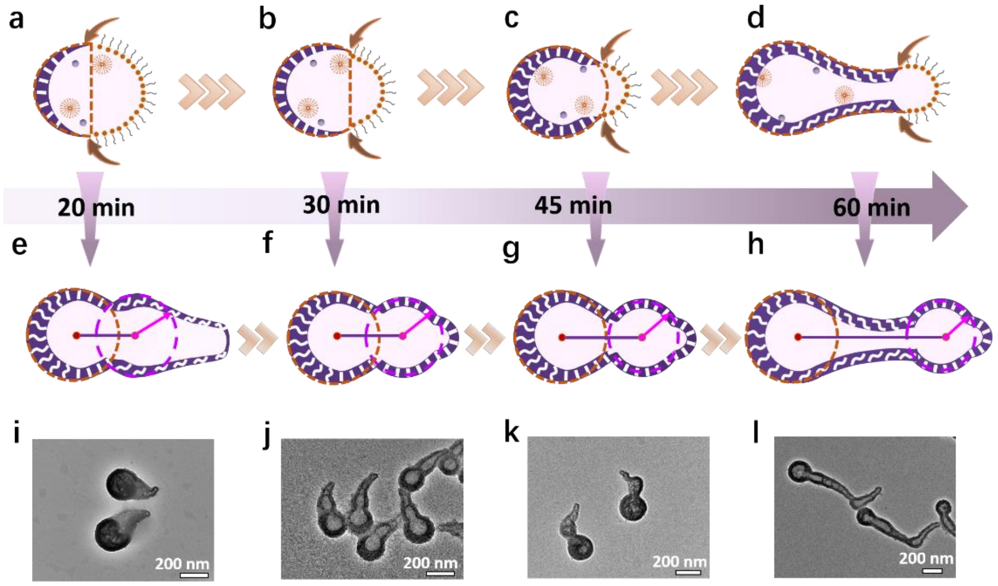

Supplementary Figure 13. Tunable chamber distribution in dual-chambered nanoparticle. (a-d) Schematic illustration of the nanodroplet remodeling at different time points. (e-h) Structural models, and (i-l) TEM images of the mesoporous silica nanoparticles with different center distance (L) obtained by regulating the time points of THF addition, (a, e, i) the 20th min, (b, f, j) the 30th min, (c, g, k) the 45th min, and (d, h, l) the 60th min. On the premise of reshaping the nanodroplets only once, adding THF prematurely results in a reduced L between the two adjacent chambers (a, e, i). In contrast, the delay of THF addition can induce the increase of L (b, f, j and c, g, k). Besides, adding THF after the first section grows to a longer neck can form an extra ellipsoidal node at the far end of the neck (d, h, l). By gradually delaying the reshaping time, the outer diameter of the second section gradually shrinks, but the distance between the two sections gradually increases from 200±28 to 377±17 nm.

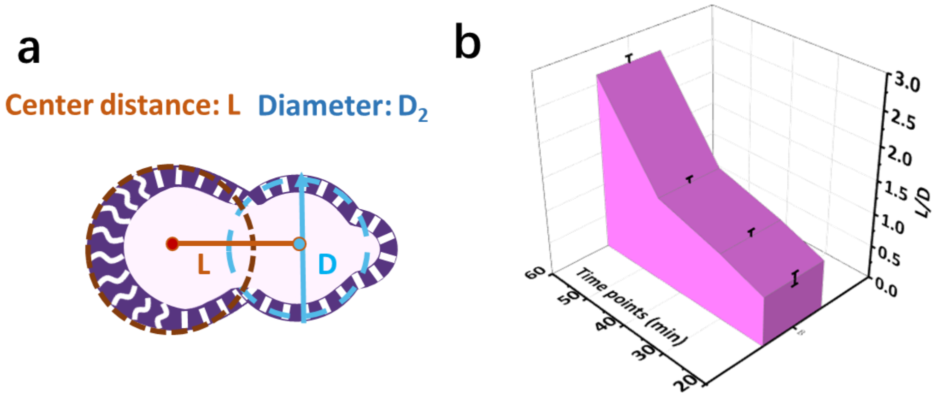

Supplementary Figure 14. Corresponding L/D values with different chamber distribution in dual-chambered nanoparticle. (a) Structural model of the mesoporous silica nanoparticle; (b) Three-dimensional diagram of the value changes of L/D at different remodeling time points; L represents the center distance between the two adjacent chambers, D is the outer diameter of the second section. It is worth noting that the L/D values gradually increases with the delay of the reshaping time. Source data are provided as a Source Data file.

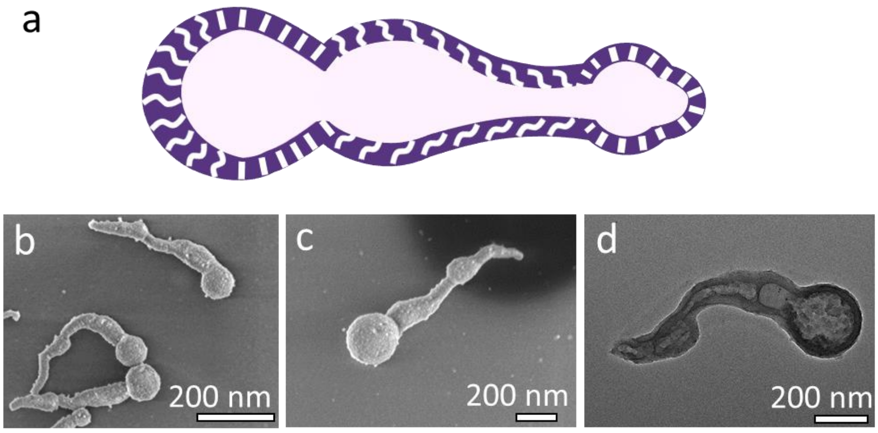

Supplementary Figure 15. Tunable chamber distribution in tri-chambered nanoparticle. (a) Structural model, (b, c) SEM, and (d) TEM images of the tri-chambered mesoporous silica nanoparticles prepared by the nanodroplet remodeling approach after adding THF twice, showing that the distribution of the third chamber can also be tuned. The center distance between the second and third chamber can further increase to 375±38 nm.

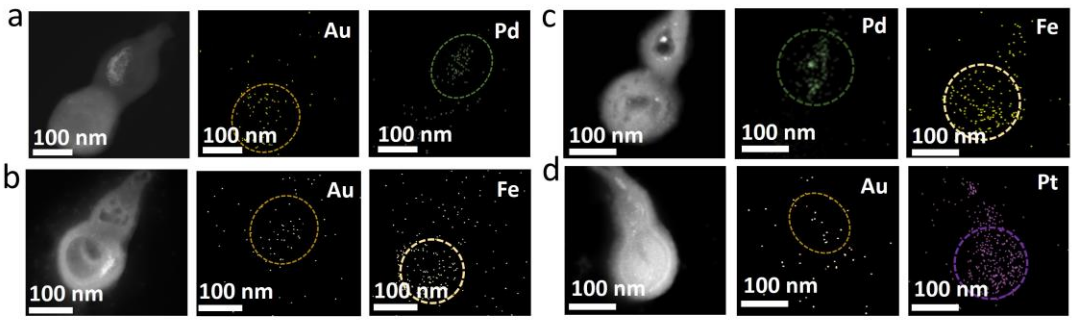

Supplementary Figure 16. Selectively in-situ assembly of different functional units. (a-d) Element mappings of the dual-chambered mesoporous silica nanoparticles with different functional units (Pt, Fe_3O_4, Pd, Au) anchored in different spatial regions. (a) Au nanocrystals (~6 nm) anchored in the bottom cavity and Pd (~4 nm) nanocrystals anchored in the upper cavity, (b) Fe_3O_4 nanoparticles (~20 nm) embedded in the bottom cavity and Au nanocrystals (~6 nm) mounted in the upper cavity, (c) Fe_3O_4 nanoparticles (~20 nm) anchored in the bottom cavity and Pd nanocrystals (~4 nm) loaded in the upper cavity, (d) Pt nanocrystals (~4 nm) embedded in the bottom cavity and Au nanocrystals (~6 nm) anchored in the upper cavity. It can be seen that the units with different functions are distributed in separate spatial regions of the obtained nanoparticles.

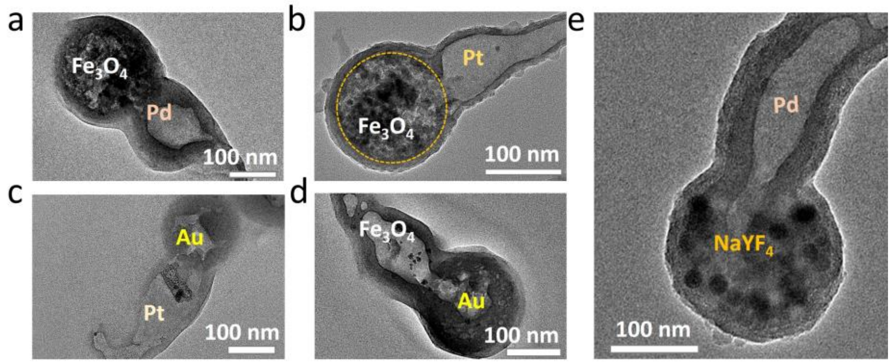

Supplementary Figure 17. Selectively in-situ assembly of different functional units. (a-e) TEM images of selective loading of two different functional units in specific locations of the dual-chambered silica nanoparticles: (a) Magnetic Fe_3O_4 nanoparticles (~20 nm) embedded in the bottom cavity and Pd nanocrystals (~4 nm) anchored in the upper cavity, (b) Fe_3O_4 nanoparticles (~20 nm) embedded in the bottom cavity and Pt nanocrystals (~4 nm) mounted in the top cavity, (c) Au nanocrystals (~6 nm) anchored in the bottom cavity and Pt nanocrystals (~4 nm) mounted in the top cavity, (d) Au nanocrystals (~6 nm) anchored in the bottom cavity and Fe_3O_4 (~20 nm) mounted in the top cavity, (e) up-conversion $NaYF_4$ nanoparticles (~22 nm) loaded in the bottom cavity and Pd nanocrystals (~4 nm) anchored in the top cavity.

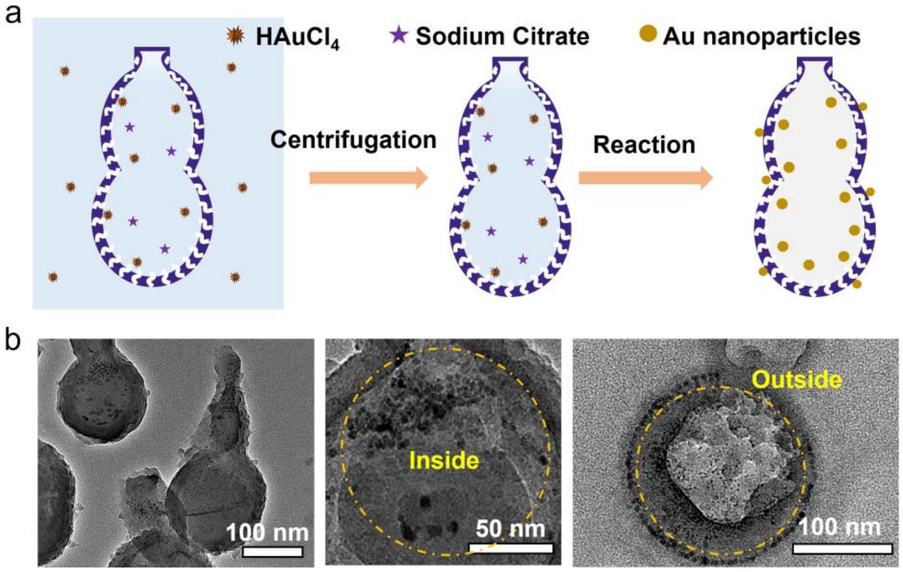

Supplementary Figure 18. In-situ preparation of Au nanoparticles in dual-chambered calabash. Illustration (a) of the *in-situ* preparation of Au nanocrystals and TEM images (b) of Au nanocrystals *in situ* prepared both on the inner and outer surfaces. Due to the presence of abundant mesopores of the nanoparticles, functional nanoparticles (such as Au) can be *in situ* fabricated at both inside and outside of the chamber.

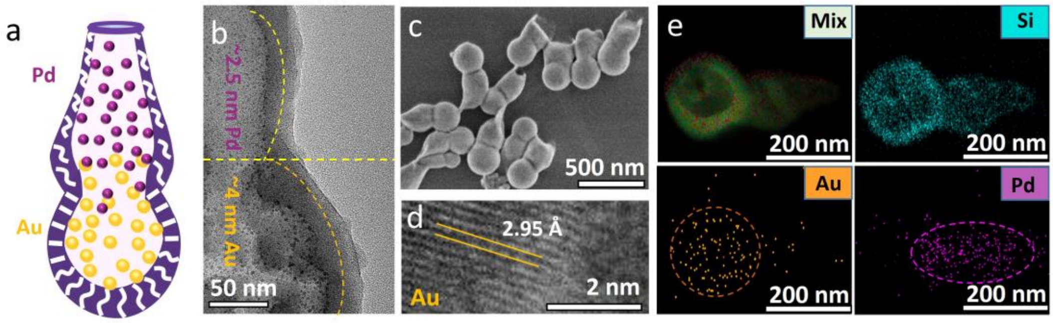

Supplementary Figure 19. Dual-chambered nanoreactors. (a) Structural model, (b, d) TEM, (c) SEM, and (e) element mapping images of the dual-chambered mesoporous silica nanoparticles as the nanoreactor with the Au nanocrystals (~4 nm) loaded in the bottom and the Pd nanocrystals (~2.5 nm) anchored in the upper opened cavity. Obviously, the Pd and Au nanocrystals are selectively loaded in different spatial regions, and the two kinds of nanocrystals are evenly embedded in the shells without excessive crosstalk.

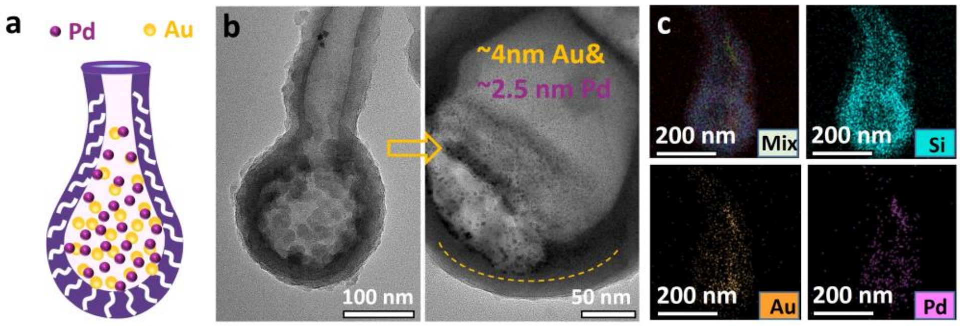

Supplementary Figure 20. Single-chambered nanoreactors. (a) Structural model, (b) TEM, and (c) element mapping images of the single-chambered mesoporous silica nanoparticles as the nanoreactor with Au (~4 nm) and Pd nanocrystals (~2.5 nm) loaded throughout the whole cavity. Visibly, a certain agglomeration is happened when the Pd nanocrystals and Au nanoparticles are loaded into the same chamber, leading to the burial of the active sites of the catalysts.

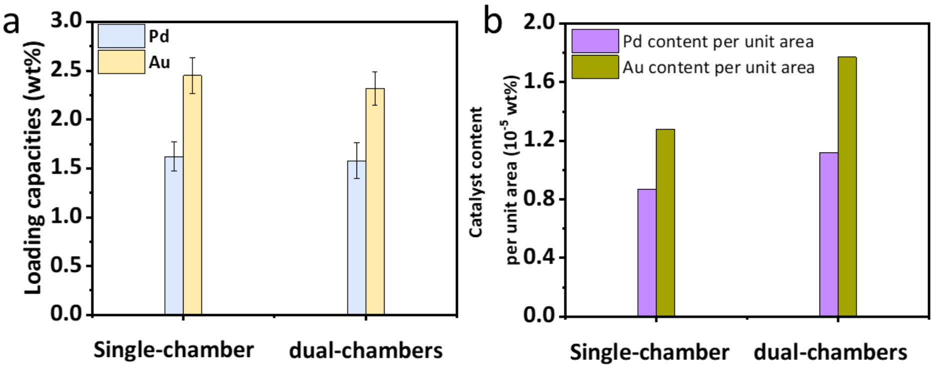

Supplementary Figure 21. Loading capacities of Pd and Au nanocrystals in different nanoreactors. (a) The loading capacities of Pd and Au nanocrystals, and (b) percentage content of Pd (Au) nanocrystals per unit area in the single- and dual-chambered nanoreactors, respectively. The spatial isolation of Pd and Au catalysts in the dual-chambered nanoparticles is achieved by a stepwise *in-situ* loading strategy. It can be seen that the loading amounts of Pd (Au) nanocrystals in the two types of nanoparticles are nearly the same. Meanwhile, the cavity volumes of single- and dual-chambered nanoparticles used in catalytic reactions are approximately the same. Therefore, although the same amount of catalysts is loaded, the separation and implantation of different nanocrystals in the dual-chambered nanoparticles result in a relatively higher concentration of catalyst per unit area, which is beneficial to the rapid catalytic reaction. Error bars represent standard deviation. Source data are provided as a Source Data file.

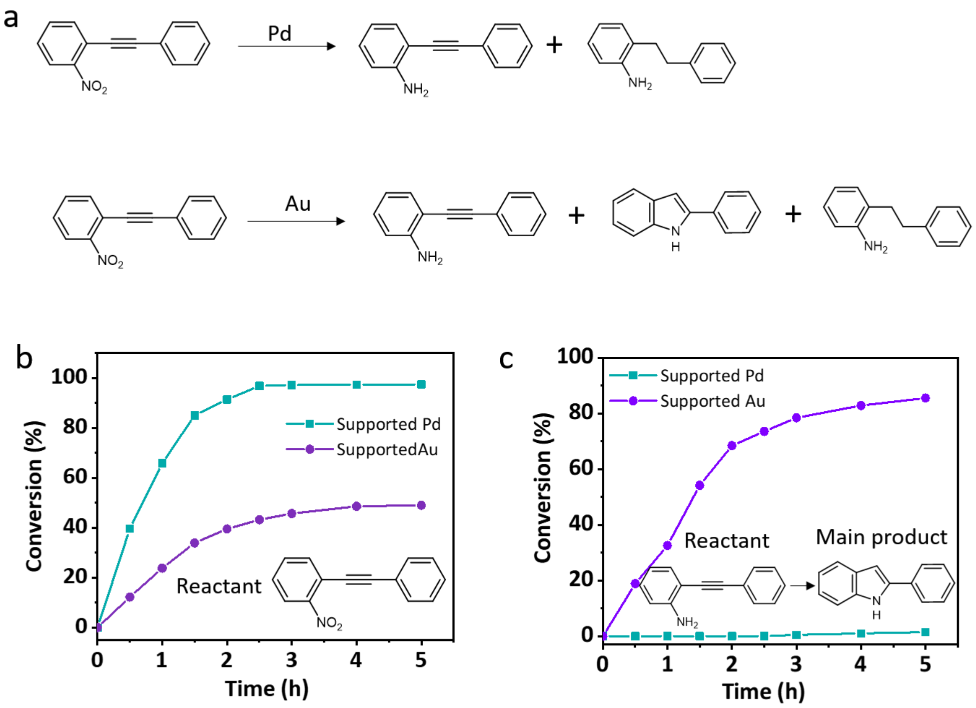

Supplementary Figure 22. Catalytic performance of monometallic catalysts in dual-chambered nanoreactors. (a) The routes of the catalytic reaction under the catalysis of supported monometallic catalysts, *i.e.*, Pd in the dual-chambered silica (**Catalyst 1**), Au in the dual-chambered silica (**Catalyst 2**); (b) Conversion efficiency of the reduction reaction (first step), and (c) the cyclization reaction (second step) under the catalysis of **Catalyst 1** and **2**, respectively. Reaction conditions: 1-nitro-2-(phenylethynyl)benzene (0.50 mmol), catalyst (Pd ~ 1.65 wt%, Au ~ 2.45 wt%), H_2 (1.5 MPa), 80 °C.

It is found that the supported monometallic Pd catalyst can give reactant 1-nitro-2-(phenylethynyl)benzene in ~100% conversion, and almost no final product 2-phenylindole can be detected, reflecting that the Pd is exclusively active in the hydrogenation reaction. On the contrary, the supported monometallic Au catalyst provides only ~48.2% conversion of 1-nitro-2-(phenylethynyl)benzene reagent, whereas the conversion of 2-(phenylethynyl)aniline to 2-phenylindole target product reaches to ~90%, demonstrating Au is mainly active for the cyclization reaction. The difference is that under the same reaction conditions, the first step is relatively faster than the second step. Source data are provided as a Source Data file.

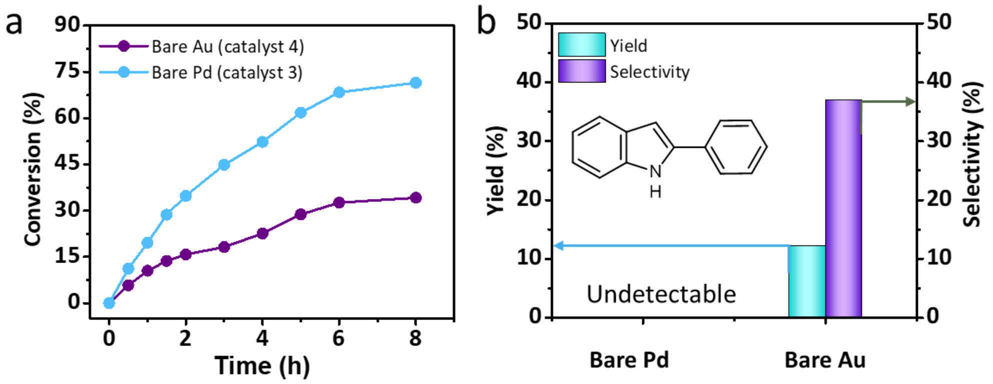

Supplementary Figure 23. Catalytic performance of monometallic bare Au/Pd catalysts. (a) Conversion efficiency of the reduction reaction, and (b) the yield and selectivity of 2-phenylindole product under the catalysis of bare Pd (**Catalyst 3**) and bare Au (**Catalyst 4**) nanocrystals, respectively. Reaction conditions: 1-nitro-2-(phenylethynyl)benzene (0.50 mmol), H_2 (1.5 MPa), 80 °C, Pd ~ 1.60 wt%, Au ~ 2.50 wt%. Similarly, the bare monometallic Pd is exclusively active in the hydrogenation reaction, while bare Au is mainly active for the cyclization reaction. Moreover, the conversion of 1-nitro-2-(phenylethynyl)benzene reagent and the yield of 2-phenylindole with bare Au/Pd are lower than that of supported catalysts, indicating the necessity of the existence of the support. Source data are provided as a Source Data file.

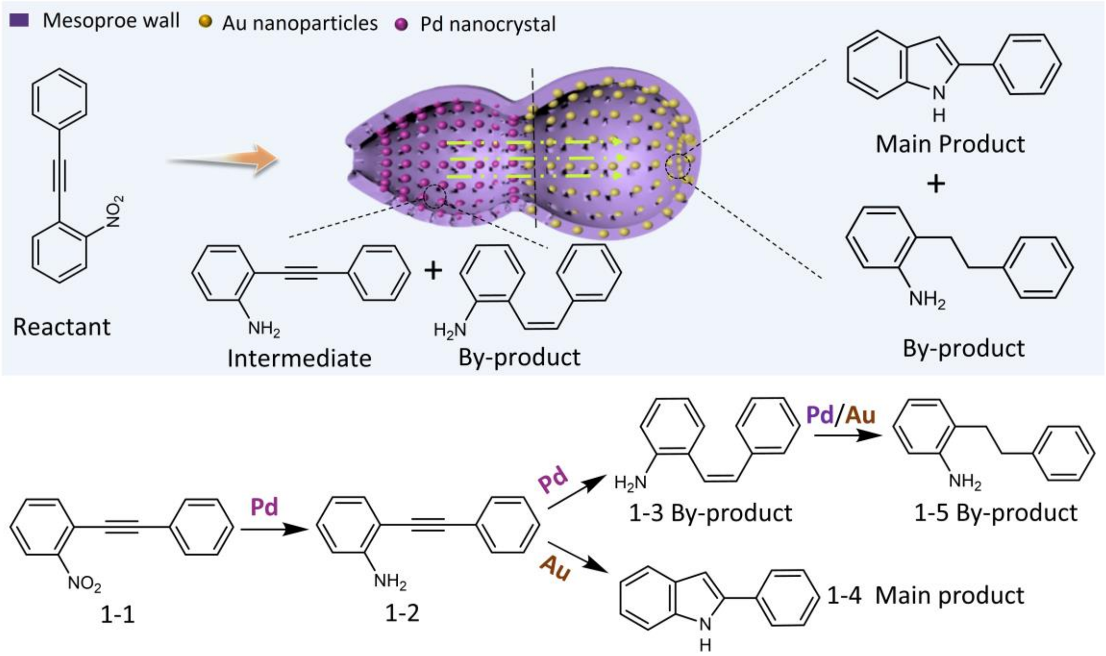

Supplementary Figure 24. Catalytic reaction in dual-chambered nanoreactors with the spatial isolation of Au and Pd catalysts. Structural model and schematic illustration of the possible by-products during the catalytic cascade synthesis of 2-phenylindole. In the dual-chambered mesoporous nanoreactors, the 1-nitro-2-(phenylethynyl)benzene reactants can be quickly converted into intermediate 2-(phenylethynyl)aniline intermediates under the catalysis of Pd nanocrystals loaded in the upper cavity. Meanwhile, a small amount of 2-styrylaniline by-products are produced with the increase of the intermediate concentration. However, due to the spatial isolation of two active centers, the 2-styrylaniline by-products can be directly transformed into 2-phenethylaniline by-products under the catalysis of Pd, which limits its competition with intermediates for the Au active sites, thus improving the selectivity to the target 2-phenylindole product. Reaction conditions: 1-nitro-2-(phenylethynyl)benzene (0.50 mmol), H_2 (1.5 MPa), 80 °C.

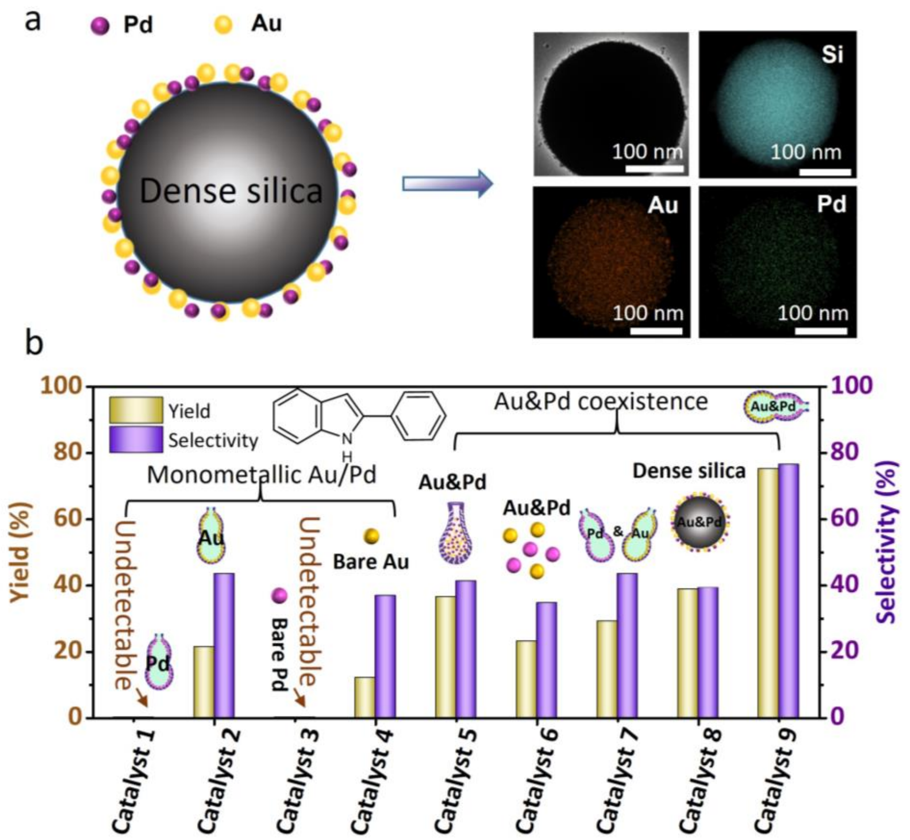

Supplementary Figure 25. Comparation of different catalysts. (a) Structural model and element mappings of the Au and Pd co-loaded dense silica sphere. (b) The yield and selectivity of 2-phenylindole under the catalysis of different catalysts. **Catalysts 1-4** are monometallic Au/Pd. **Catalyst 1**: Pd in the dual-chambered silica; **Catalyst 2**: Au in the dual-chambered silica; **Catalyst 3**: bare Pd nanocrystals; **Catalyst 4**: bare Au nanocrystals. **Catalysts 5-9** are the co-existence of Au and Pd. **Catalyst 5**: mixed-Au and Pd in single-chambered silica particles; **Catalyst 6**: mixed-Au and Pd nanocrystals; **Catalyst 7**: mixed-catalyst 1 and 2; **Catalyst 8**: mixed-Au and Pd on dense silica nanoparticles; **Catalyst 9**: spatial separation of Au and Pd in dual-chambered silica particles. Reaction conditions: 1-nitro-2-(phenylethynyl)benzene (0.50 mmol), H_2 (1.5 MPa), 80 °C.

Similarly, both mixed-catalyst 1 and 2 (**Catalyst 7**, 29.2% yield of target product), and Au-Pd mixed-loaded dense silica (**Catalyst 8**, 38.9% yield of target product) catalysts can also increase the yield of the target product to a certain extent (Figure S25), but the by-products are also increased simultaneously, resulting in the selectivity remaining basically unchanged compared with that of Au and Pd co-stored single-chambered catalyst (Table S2). These results are most likely due to the fact that the Pd and Au active centers are either too far apart (**Catalyst 6** and **7**) or excessively cross-talk (**Catalyst 5** and **8**), resulting in few intermediates that can diffuse to the independent Au active sites, thus increasing the production of by-products. Source data are provided as a Source Data file.

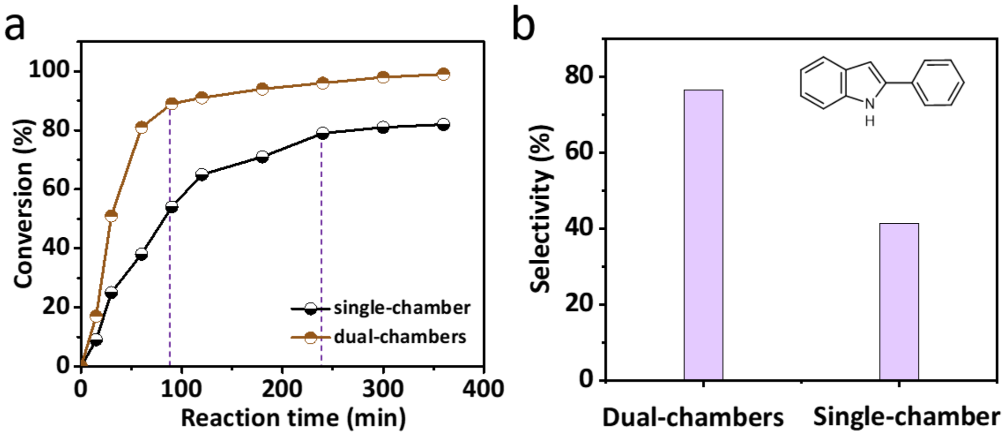

Supplementary Figure 26. Comparation of catalytic performance of different nanoreactors with Au and Pd catalysts. (a) Conversion efficiency of the 1-nitro-2-(phenylethynyl)benzene in different nanoreactors loaded with Pd and Au catalysts. (b) The selectivity to 2-phenylindole product in nanoreactors with different chamber structures. Reaction conditions: 1-nitro-2-(phenylethynyl)benzene (0.50 mmol), H_2 (1.5 MPa), 80 °C.

It can be seen that the conversion rate exceeds 90% within 2 h by using the dual-chambered nanoparticles as a nanoreactor, implying that the first step is almost complete. In contrast, the conversion rate only reaches to ~80% after 4 h when the single-chambered nanoparticles are used as the nanoreactor, further demonstrating that the spatial isolation of catalytic active centers is beneficial to improve the reaction efficiency. Meanwhile, the spatial isolation of active centers greatly improves the selectivity to 2-phenylindole product, which increases from 41.3% (single-chamber) to 76.5% (dual-chambers). Source data are provided as a Source Data file.

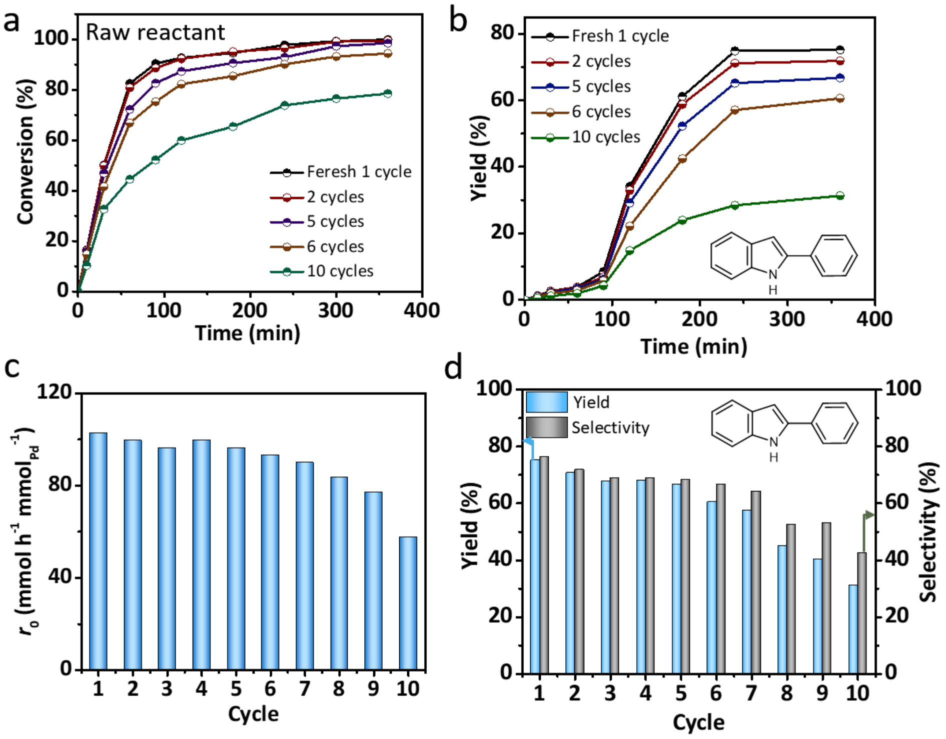

Supplementary Figure 27. Catalytic cycle stability tests. (a) The conversion plots, (b) yield curves, (c) comparison of the initial reaction rates, and (d) cycling stability of the Au and Pd in the dual-chambered nanoreactors at different cycles. The initial reaction rate was calculated at the beginning (at a conversion of less than 20%) of the reaction (mmol of reacted 1-nitro-2-phenylethynylbenzene)/(mmol of Pd •h). The recycling test showed that the conversion-time curves of the fresh and second reused catalysts were almost coincide, and both the conversion and the yield exhibited relatively slow decay rates in the first five cycles. However, the decay rate was accelerated after five cycles, the conversion efficiency decreased to ~78.5% after ten cycles and the yield of the target product was only 31.3%. The same trend was observed for the initial reaction rates. The corresponding initial reaction rates of the first five cycles decayed slowly from ~ 102.78 for the first cycle to ~ 99.57 mmol h^{-1} mmol$_{pd}^{-1}$ for the fifth cycle. However, further increasing to 10 cycles, the initial reaction rate was reduced to 57.81 mmol h^{-1} mmol$_{pd}^{-1}$. Source data are provided as a Source Data file.

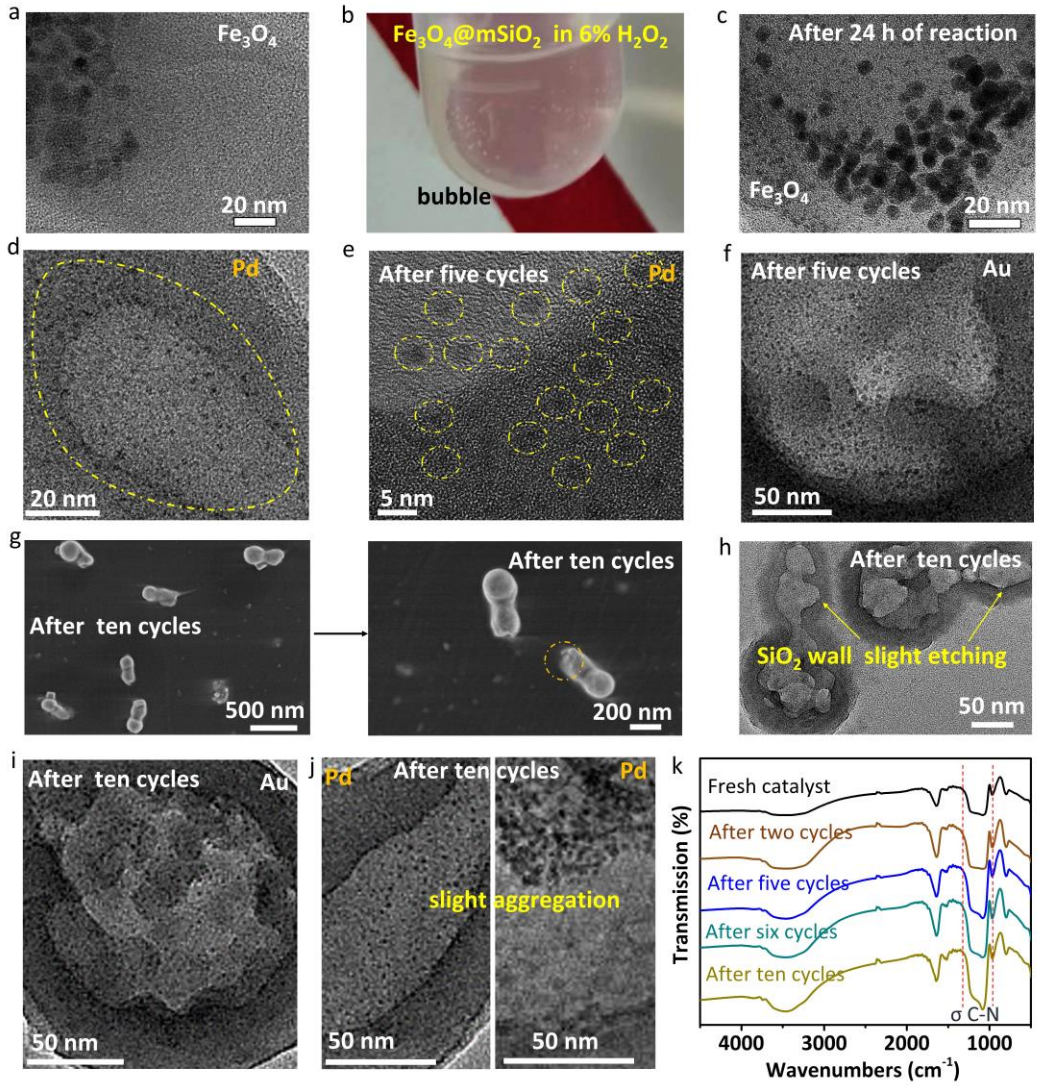

Supplementary Figure 28. Characterization of the catalysts after being reused different cycles. (a, c) TEM images of the Fe_3O_4-loaded nanoparticles; (b) The optical photo of the H_2O_2 solution (6%) after adding the Fe_3O_4-loaded nanoparticles. The Fe_3O_4 were implanted in the silica shells of the nanoparticles. After adding the Fe_3O_4-loaded nanoparticles into the H_2O_2 solution (6%), the bubbles could be clearly observed. Even after 24 h of reaction, the Fe_3O_4 nanoparticles were still well anchored at the head part of the cavity, further illustrating a high stability of the Fe_3O_4 nanoparticles in the cavity. (d-i) TEM images with different magnifications of the Pd and Au loaded nanoparticles before and after the catalytic reactions: (d) before the catalytic reaction, after (e, f) five and (g-j) ten cycles of catalytic reaction. After five rounds of catalytic reactions, Pd/Au was still well anchored in the cavity, and no obvious Pd/Au leaching was detected in the reaction solution. However, a very small amount of Pd leaching (~ 0.11%) was detected after the tenth cycle, which might be caused by the slight etching effect of the catalytic reactions on the inner walls of the SiO_2 support. (k) FT-IR spectra of the reused catalysts after different cycles; The FT-IR spectra demonstrated that some nitrogenous species were adsorbed onto the reused catalyst, especially after ten cycles, which might lead to the poisoning of the catalyst. Source data are provided as a Source Data file.

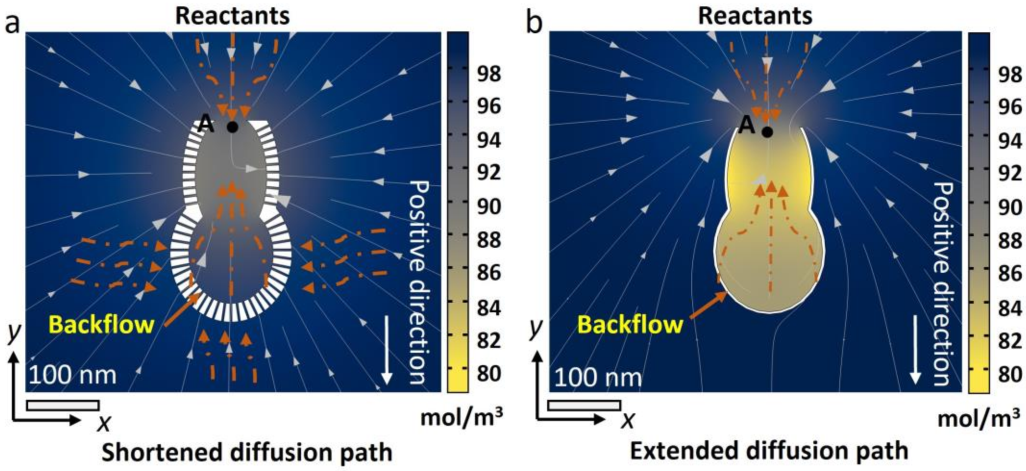

Supplementary Figure 29. The simulated transient-state concentration gradient distribution of the reactants in the dual-chambered nanoreactors with or without mesopores on the shell at 20 μs. (a) Mesoporous shells, (b) non-porous shells. Finite element analysis was used to evaluate the transient-state concentration gradient distribution of the intermediate products in the chambers. The simulations were performed in a time-dependent mode at a microsecond scale due to the rapid evolution of physical fields. Unlike non-porous dual-chambered nanoreactors, in which reactants can only enter the cavity through the opening. The external reactants can not only enter the cavity through the opening of the mesoporous nanoreactors, but also directly re-diffuse into the chamber through the mesopore shells, which can effectively shorten the diffusion path of molecules.

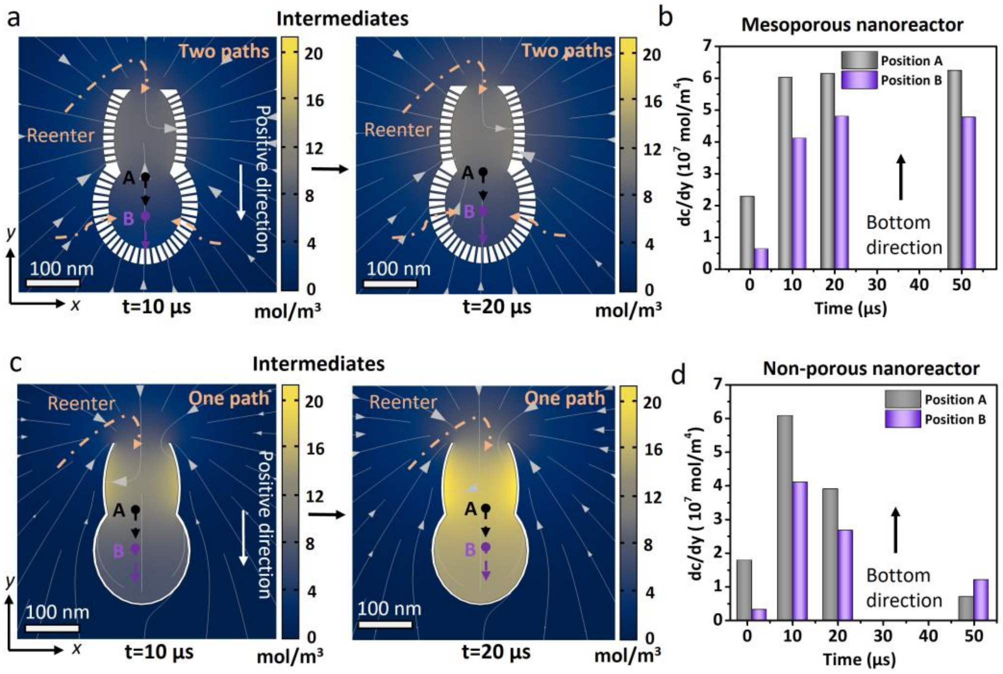

Supplementary Figure 30. The simulated transient-state concentration gradient distribution of the intermediates in the dual-chambered nanoreactors with or without mesopores on the shell at different times. (a, c) The simulated transient-state concentration gradient distribution, and (b, d) corresponding calculated transient-state concentration gradient of the intermediates at A and B positions in dual-chambered nanoreactors with or without mesopores on the shell at 10 and 20 µs, respectively. A is the middle position of the whole cavity, and B is middle location of the bottom chamber. (a, b) Mesoporous shells, (c, d) non-porous shells. It can be seen that in both nanoreactors, the 2-(phenylethynyl)aniline intermediate is initially produced in the upper chamber, and then a local concentration gradient is generated at the inner cavity of the dual-chambered nanoreactor. Driven by the resultant concentration gradient, a high diffusion flux is rapidly generated, thereby enabling the sustained and efficient diffusion of intermediates to the bottom chamber. However, compared with the non-porous nanoreactor, the existence of mesopores makes it possible to maintain a relatively higher diffusion flux at A and B positions, reflecting the enhanced diffusion to bottom chamber, which facilitates the subsequent cyclization to produce 2-phenylindole. More importantly, for the non-porous dual-chambered nanoreactors, the intermediates diffused to the outside can only reenter the cavity from the opening, which inevitably contacts the Pd nanoparticles in the upper cavity, thus increasing the probability of by-product formation. It is not conducive to improve the selectivity of target product 2-phenylindole. On the contrary, the external intermediates can re-diffuse into the bottom chamber with Au active sites through the mesoporous shells, and the internal intermediates can also maintain a high diffusion flux to the bottom chamber, both of which are crucial to improve the selectivity of the target product. Source data are provided as a Source Data file.

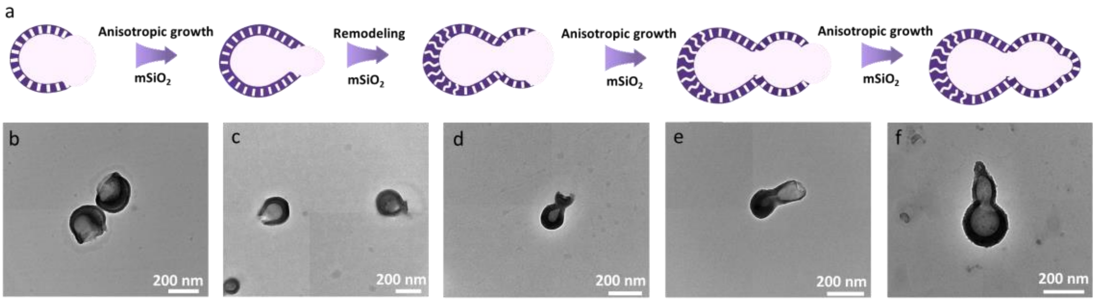

Supplementary Figure 31. The formation process of dual-chambered nanoparticles. (a) Schematic illustration and (b-f) TEM images of the intermediate products of the mesoporous silica nanoparticles obtained at different time intervals: (b) 20 min; (c) 30 min; (d) 45 min; (e) 60 min; (f) 120 min. It could be seen that the thin silica shells were formed within 0.5 h. With the increase of the reaction time, the opening size of the silica nanoparticles decreased gradually. With the addition of THF solvent, the THF diffuses into the water phase and causes volume expansion, which produces an abrupt turn in the streamlined nanoparticles, resulting in an extra streamlined node. When the reaction time was extended to 2 h, an enclosed dual-chambered structure could finally be formed. The growth of the second segment is very similar to that of the first, except that the second section becomes smaller.

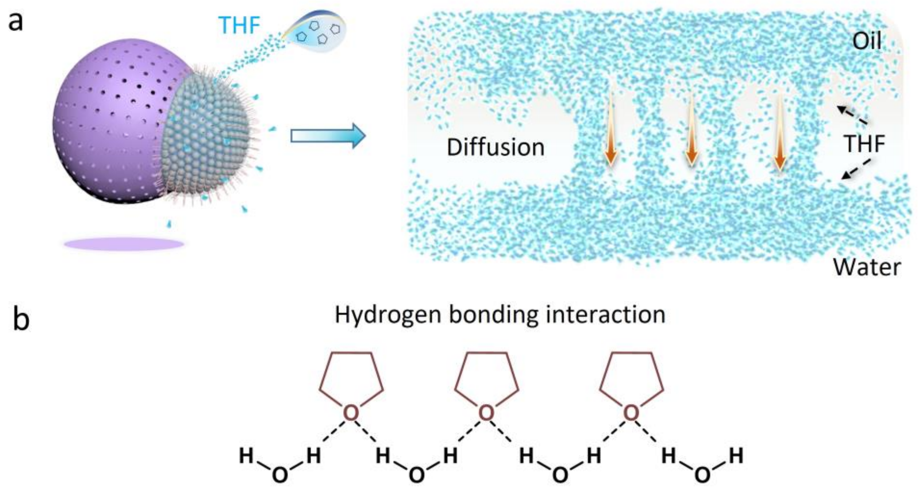

Supplementary Figure 32. The driving force of THF diffusion. (a) Schematic diagram of the diffusion of THF from the oil to the water phase, (b) the intermolecular hydrogen bonding between THF and water molecules. After the addition of THF, partial THF molecules diffuse into the water phase driven by the intermolecular hydrogen bond interactions, which further results in the volume expansion of the water nanodroplets.

Supplementary Tables

Supplementary Table 1. Oil-water distribution coefficients of different solvents in pentanol-water system.

Solvent	EtOH	Propanol	Butanol	Hexanol	Octanol	THF	Hexane	DMF
K_{ow}	-0.14	0.25	0.88	1.82	2.03	0.94	3.90	-0.93

Supplementary Table 2. One-pot synthesis of 2-phenylindole by supported Pd and Au catalysts under hydrogenation condition.

Number	Cat.	Convn. [a] %	Yield [a] %		
			1-2	1-4	1-3+1-5
Catalyst 1	[Pd]	>99	63.8	0	35.4
Catalyst 2	[Au]	48.2	15.9	22.4	11.8
Catalyst 3	Pd	71.5	41.6	0	28.2
Catalyst 4	Au	34.2	13.7	12.2	7.1
Catalyst 5	[Pd and Au]	88.9	25.4	36.6	26.5
Catalyst 6	[Pd and Au]	85.4	30.3	27.2	26.8
Catalyst 7	Pd and Au	67.3	26.2	23.1	17.1
Catalyst 8	[Pd and Au]	96.6	26.4	38.7	31.5
Catalyst 9	[Pd and Au]	99.2	12.2	75.2	11.9

Reaction conditions: nitroarenes (0.50 mmol), catalyst (Pd ~1.65 wt%, Au 2.45 wt%), ethanol (5.0 mL), H_2 (1.5 MPa), 80 °C.

[a]Detected by GC-MS.

[] represent silica support.

Supplementary references

1. Cui, M., Emrick, T., Russell, T. P. Stabilizing liquid drops in nonequilibrium shapes by the interfacial jamming of nanoparticles. *Science* **342**, 460-463 (2013).
2. Thompson, K. L., Fielding, L. A., Mykhaylyk, O. O., Lane, J. A., Derrya, M. J., Armes, S. P. Vermicious thermo-responsive Pickering emulsifiers. *Chem. Sci.*, **6**, 4207-4214 (2015).
3. Wu, J., Ma, G. H. Recent studies of Pickering emulsions: particles make the difference. *Small* **34**, 4633-4648 (2016).
