# Supplementary material for: Remodeling nanodroplets into hierarchical mesoporous silica nanoreactors with multiple chambers
Source: Nat Commun. 2022 Oct 17;13:6136. doi: 10.1038/s41467-022-33856-y (PMC9576742; doi:10.1038/s41467-022-33856-y)
Supplement: Supplementary file 2 — Description of Additional Supplementary Files [file 41467_2022_33856_MOESM2_ESM.pdf]

Supplementary Movie 1: reactants in dual-chambered mesoporous nanoreactor.

Supplementary Movie 2: reactants in single-chambered mesoporous nanoreactor.

Supplementary Movie 3: intermediates in dual-chambered mesoporous nanoreactor.
