# Supplementary material for: Remodeling nanodroplets into hierarchical mesoporous silica nanoreactors with multiple chambers
Source: Nat Commun. 2022 Oct 17;13:6136. doi: 10.1038/s41467-022-33856-y (PMC9576742; doi:10.1038/s41467-022-33856-y)
Supplement: Supplementary file 6 — Reporting Summary [file 41467_2022_33856_MOESM6_ESM.pdf]

nature portfolio

Corresponding author(s): Dongyuan Zhao

Last updated by author(s): Sep 29, 2022

Reporting Summary

Nature Portfolio wishes to improve the reproducibility of the work that we publish. This form provides structure for consistency and transparency in reporting. For further information on Nature Portfolio policies, see our Editorial Policies and the Editorial Policy Checklist.

Statistics

For all statistical analyses, confirm that the following items are present in the figure legend, table legend, main text, or Methods section.

n/a	Confirmed	
☐	☒	The exact sample size (*n*) for each experimental group/condition, given as a discrete number and unit of measurement
☐	☒	A statement on whether measurements were taken from distinct samples or whether the same sample was measured repeatedly
☐	☒	The statistical test(s) used AND whether they are one- or two-sided<br>*Only common tests should be described solely by name; describe more complex techniques in the Methods section.*
☒	☐	A description of all covariates tested
☒	☐	A description of any assumptions or corrections, such as tests of normality and adjustment for multiple comparisons
☐	☒	A full description of the statistical parameters including central tendency (e.g. means) or other basic estimates (e.g. regression coefficient) AND variation (e.g. standard deviation) or associated estimates of uncertainty (e.g. confidence intervals)
☒	☐	For null hypothesis testing, the test statistic (e.g. *F*, *t*, *r*) with confidence intervals, effect sizes, degrees of freedom and *P* value noted<br>*Give P values as exact values whenever suitable.*
☒	☐	For Bayesian analysis, information on the choice of priors and Markov chain Monte Carlo settings
☐	☒	For hierarchical and complex designs, identification of the appropriate level for tests and full reporting of outcomes
☒	☐	Estimates of effect sizes (e.g. Cohen's *d*, Pearson's *r*), indicating how they were calculated

Our web collection on statistics for biologists contains articles on many of the points above.

Software and code

Policy information about availability of computer code

Data collection	COMSOL Multiphysics software was used to simulate the transient-state concentration gradient distribution of products.
Data analysis	COMSOL Multiphysics software was used to simulate the transient-state concentration gradient distribution of products.<br>Image management: Adobe Photoshop CS6;<br>Data representation: OriginPro 8.0, Microsoft Powerpoint 2016, 3D Studio Max 2018.

For manuscripts utilizing custom algorithms or software that are central to the research but not yet described in published literature, software must be made available to editors and reviewers. We strongly encourage code deposition in a community repository (e.g. GitHub). See the Nature Portfolio guidelines for submitting code & software for further information.

Data

Policy information about availability of data

All manuscripts must include a data availability statement. This statement should provide the following information, where applicable:

- Accession codes, unique identifiers, or web links for publicly available datasets
- A description of any restrictions on data availability
- For clinical datasets or third party data, please ensure that the statement adheres to our policy

Data supporting the findings of this study are available within the article and the associated Supplementary Information Section. The source data underlying Fig. 1-6 and Supplementary Figs. are provided in a Source Data file. Source data are provided with this paper.

Human research participants

Policy information about studies involving human research participants and Sex and Gender in Research.

Reporting on sex and gender	Human research participants and Sex and Gender in research were not included in this study.
Population characteristics	See above
Recruitment	See above
Ethics oversight	See above

Note that full information on the approval of the study protocol must also be provided in the manuscript.

Field-specific reporting

Please select the one below that is the best fit for your research. If you are not sure, read the appropriate sections before making your selection.

☐ Life sciences ☐ Behavioural & social sciences ☒ Ecological, evolutionary & environmental sciences

For a reference copy of the document with all sections, see nature.com/documents/nr-reporting-summary-flat.pdf

Ecological, evolutionary & environmental sciences study design

All studies must disclose on these points even when the disclosure is negative.

Study description	We reported a nanodroplet remodeling strategy for fabrication of hierarchical multi-chambered mesoporous silica nanoparticles with tunable chamber numbers.
Research sample	The research sample is mainly focused on the dual-chambered nano-particles.
Sampling strategy	More than ten locations were randomly selected on the SEM/TEM grid to analyze the obtained products. We counted more than 500 particles and estimated the structural parameters (e.g., body length).
Data collection	Y.A., B.M., K.L., and C.W., assisted Y.M. for the synthesis of materials and the data collection and analysis, H.Z., W.C., X.D. and L.D., involved in partial nanoreactor data and analysis. Nitrogen adsorption-desorption isotherms were performed at 77 K with a Micromeritcs Tristar 3020 analyzer (USA).The field-emission scanning electron microscope (FESEM) observations were taken on a Hitachi FE-SEM-4800 microscope operating at 20 kV without any metal coating. Transmission electron microscopy (TEM) measurements were taken on a JEOL JEM-2100F microscope (Japan) operated at 200 kV. Catalytic data were obtained by GC-MS.
Timing and spatial scale	The start and stop dates of data collection were Jul. 17, 2021 and Aug. 17, 2022, respectively. From product preparation to characterization of physical properties and the subsequent application testing, the overall frequency and periodicity of sampling were about once a month.
Data exclusions	No data were excluded from the analyses.
Reproducibility	By scaling up the reaction, or by changing different droplet expansion agents to obtain dual-chambered nano-particles. Exact numbers for each individual type of experiment are provided in Figure legends and in the Source Data file. All attempts to repeat the experiment were successful.
Randomization	Solutions containing nano-particles with different chamber numbers were randomly added dropwise onto the Cu grids. The corresponding particles were randomly selected in SEM/TEM grids.
Blinding	Investigators were not blinded to group allocation during data collection and analysis due to limited operators and funds budget.

Did the study involve field work? ☐ Yes ☒ No

Reporting for specific materials, systems and methods

We require information from authors about some types of materials, experimental systems and methods used in many studies. Here, indicate whether each material, system or method listed is relevant to your study. If you are not sure if a list item applies to your research, read the appropriate section before selecting a response.

Materials & experimental systems

n/a	Involved in the study
☒	☐ Antibodies
☒	☐ Eukaryotic cell lines
☒	☐ Palaeontology and archaeology
☒	☐ Animals and other organisms
☒	☐ Clinical data
☒	☐ Dual use research of concern

Methods

n/a	Involved in the study
☒	☐ ChIP-seq
☒	☐ Flow cytometry
☒	☐ MRI-based neuroimaging
